# Spatial–temporal patterns, seasonality, and age-specific trends of varicella in Hangzhou, China, 2019–2024

**DOI:** 10.3389/fpubh.2026.1701894

**Published:** 2026-01-30

**Authors:** Lintao Gu, Yan Liu, Xiaoping Zhang, Yuyang Xu, Xuechao Zhang, Xinren Che, Wenwen Gu, Yingying Yang, Lu Zj

**Affiliations:** Hangzhou Center for Disease Control and Prevention (Hangzhou Health Supervision Institution), Hangzhou, China

**Keywords:** age-specific trends, harmonic regression model, seasonality, spatial analysis, varicella (chickenpox)

## Abstract

**Background:**

Varicella has been subject to mandatory reporting to the China Information System for Disease Control and Prevention (CISDCP) by health agencies within 24 h of diagnosis since 2019. However, even if two-dose varicella vaccination has been recommended to be administered to children at 1 and 4 years of age in Hangzhou since 2014, emerging evidence of increasing breakthrough varicella cases in outbreaks challenges the present varicella vaccination schedule and its protective effect. We seek to identify hotspot areas and temporal trends of varicella at the township level in Hangzhou in the recent 6 years by using spatiotemporal analysis.

**Methodology:**

Varicella cases diagnosed by medical practitioners from 2019 to 2024, demographic data, and clinical data were extracted from CISDCP. Township-level population figures were estimated using a constant-share proportional allocation method based on the seventh census data in China. Global I statistics and the local index spatial autocorrelation (LISA) method were used to identify global autocorrelation and local autocorrelation, respectively. Retrospective spatial scan statistics were undertaken to explore potential spatiotemporal clusters of varicella. A harmonic regression model was used to quantify seasonality, and an age-specific trend was evaluated through the Cochrane-Armitage test.

**Result:**

A continuous decline in reported incidence of varicella in Hangzhou from 2019 to 2024 was observed, with 97.95 per 100,000 and 52.23 per 100,000 in 2019 and 2024, respectively. Seasonality of the bimodal peak was observed, with the first peak of varicella cases observed from May to July, whereas the second peak typically occurs from November to February of the following year. A pronounced reduction in varicella incidence among younger children (5–9) and a relatively slower decline in older pediatric and adolescent groups (10–19) were found. The spatial distribution pattern of varicella in Hangzhou at township levels was non-random, and hotspots tend to be more frequent in the suburbs than in downtown areas. A total of 34 significant varicella spatiotemporal clusters were identified by retrospective space–time scan statistics, the vast majority of which were located in suburban areas.

**Conclusion:**

Varicella incidence has dramatically declined over the past 6 years. The 10–19-year-old age band exhibited a slower reduction than the 5–9-year-old age-band. Moreover, the tendency for varicella clusters to appear more frequently in suburban areas reflects disparities in varicella incidence geographically. Specific surveillance and control measures should be undertaken in high-incidence regions in Hangzhou.

## Introduction

Varicella is a respiratory contagious disease caused by the varicella-zoster virus (VZV), clinically manifested as generalized itchy vesicular exanthema, generally accompanied by fever and appetite loss (1), remains a persistent public health concern despite the widespread implementation of effective vaccination programs (2, 3). Although generally self-limiting in children, the disease is highly contagious and can result in severe complications, including encephalitis, pneumonitis, secondary bacterial infections, hospitalization, or even death (4) among adults, pregnant women, and immunocompromised individuals (5). In regions with high vaccine coverage, varicella vaccination has resulted in substantial disease prevention and societal savings (6–8). Varicella has been subject to mandatory reporting to the China Information System for Disease Control and Prevention (CISDCP) by health agencies within 24 h of diagnosis since 2019, as required by the Hangzhou Bureau of Health. However, even if two-dose varicella vaccination has been recommended to be administered to children at 1 and 4 years of age in Hangzhou since 2014, it is still a non-National Immunization Program vaccine, being self-paid and administered voluntarily. Emerging evidence suggests that the actual varicella disease burden in China may be significantly underestimated due to underreporting and heterogeneous vaccine coverage geographically (9). The varicella vaccine administration schedule and its protective effect have been under debate since breakthrough cases in outbreaks have increasingly been identified in previous studies (10, 11). Low varicella vaccine coverage can facilitate the occurrence of an outbreak (12). Previous studies have established that socioeconomic characteristics play a pivotal role in the distribution of coverage for non-National-Immunization-Program vaccines (13, 14). Uneven distribution of healthcare resources may lead to geographically heterogeneous vaccine uptake, resulting in the spatial aggregation of the susceptible population. Consequently, these processes may manifest as spatial clustering of varicella cases or incidences at the town or street level (15). In this context, spatial–temporal analysis of incidence can provide critical insights into transmission dynamics, identify persistent hotspots, and inform a more targeted and effective varicella vaccination strategy (16, 17).

However, while previous studies have widely utilized spatial analysis, applying both global autocorrelation and LISA to identify varicella aggregation patterns in other regions of China, they often rely on district-level administrative surveillance data (15, 18). However, as emphasized by Haque et al. in 2025 (19), finer spatial unit spatial data can improve estimates and spatial statistic inference precision, whereas coarser spatial units may lead to greater uncertainty. Because the aggregation from district-level data may dilute the local spatial autocorrelation and obscure subtle local hotspots due to the aggregation and zoning effects of the Modifiable Areal Unit Problem (MAUP) (19, 20). Besides, although seasonal patterns of varicella have been delineated by previous researchers (18, 21), none of them have attempted to quantify seasonality using harmonic regression. To address these gaps, our study aims to characterize the spatiotemporal distribution of varicella incidence in Hangzhou at town/street granularity from 2019 to 2024, drawing on surveillance data from CISDCP. Through using spatial clustering, seasonality quantifying analysis, and age-specific trend analysis, we seek to identify high-incidence areas and temporal trends, thereby providing empirical evidence to support future vaccine planning and outbreak prevention strategies.

## Materials and methodology

### Data source and collection

Reporting varicella cases in the database of the China Information System for Disease Control and Prevention (CISDCP) with symptom onset and date from 2019 to 2024 were considered eligible for this study (22). Additionally, demographic data, including occupation, residential address, age, gender, and clinical data, including symptom onset date-time and case classification, were collected for analysis. A varicella case was defined as a patient who has acute symptoms of pruritic papule and vesicular skin rash that forms small, itchy blisters that scab over without any identifiable alternative causes. Patients whose residential address cannot be identified and classified into township levels were excluded from this study. Township-level population data in 2020 were obtained from the seventh Chinese census; population data for the remaining 5 years were estimated using a constant-share proportional allocation method based on population data of the seventh census at town/street granularity and district-level population figures for each respective year, as reported in the Hangzhou Statistical Yearbook, under the assumption that the age structure did not change dramatically at the town/street level in Hangzhou from 2019 to 2024.

### Seasonality quantification and age-specific trend assessment

We used a harmonic regression model to estimate the peak timing and strength of varicella cases in each respective town/street, as described by previous studies (23). To obtain robust estimation, an offset term was included for the monthly population to control the long-term temporal trends and changes in surveillance efforts or the size of the population under surveillance over the study period (24). A negative binomial generalized linear model was fitted after we tested both Poisson and negative binomial models, since the mean and variance estimates suggested an over-dispersion of varicella case counts. The model structure allows for simultaneous estimates of both annual and semi-annual periodicity. The negative binomial model accounting for seasonality is given by Equation (1):

Y(t) ~ Negative binominal (𝜆𝑡)


$$\begin{array}{l} \ln(\lambda_{t})=\beta_{0}+\beta_{1}\sin(\frac{2\mathrm{\pi t}}{12})+\beta_{2}\cos(\frac{2\mathrm{\pi t}}{12})+\\ \beta_{3}\sin(\frac{2\mathrm{\pi t}}{6})+\beta_{4}\cos(\frac{2\mathrm{\pi t}}{6})+\\ \beta_{5}t+\beta_{6}t^{2}+\ln(n_{t})+\varepsilon \end{array}$$ (1)

Where Y(t) is the monthly case count of varicella in each respective township and t is the running time index in months over time. The term ln(𝜆𝑡) is the logarithm of the expected case count of varicella. The log-transformed monthly number of populations ( $\ln(n_{t})$ ) in each town/street is included in the model as an offset term to normalize the case counts, allowing the coefficients in the model to be interpreted as the change in the log incidence rate instead of the absolute case count. The whole-year and half-year periodicity is denoted by the two paired trigonometric terms (where $\beta_{1}$ and $\beta_{2}$ denote primary annual seasonality, while $\beta_{3}$ and $\beta_{4}$ denote semiannual cycles.)

Seasonality was then quantified using metrics derived from model coefficients. The amplitude that indicates the annual precocity was computed as the sqrt ( $\beta_{1}$ ^2^ + $\beta_{2}$ ^2^) and semi-amplitude as the sqrt ( $\beta_{3}$ ^2^ + $\beta_{4}$ ^2^). The phase angle for the primary peak was calculated as arctan ( $\beta_{1}$ / $\beta_{2}$ ) and then converted from radians to months using the methods as described by previous researchers (25). We define the semi-periodicity contribution ratio as the ratio between semi-amplitude and the sum of semi and annual amplitude, and a ratio exceeding 0.5 was interpreted as evidence of dominant semiannual (bimodal) seasonality (24). Uncertainty (95% confidence interval) in annual seasonality strength and peak timing was assessed using a seasonal block bootstrap method with 1,000 replicates to retain the inherent autocorrelation structure of time-series data (24, 26). Age-specific trend was tested with the Cochrane-Armitage test, and the annual percent change (APC) was estimated through join-point regression modeling (27), with a *p*-value threshold of 0.05 used to determine significance.

#### Spatial autocorrelation analysis

Spatial autocorrelation refers to the relationship between adjacent spatial units, which quantifies the degree to which the interested geographical features and their relevant values tend to be clustered (positively correlated) or dispersed (negatively correlated) or random (28). In this study, the global Moran’s I statistic was used to evaluate global spatial autocorrelation. It is computed by Equation (2):


$$I=\frac{n}{\sum_{i}\sum_{j}w_{\mathrm{ij}}}*(\sum_{i}\sum_{j}w_{\mathrm{ij}}(x_{i}-\bar{x})(x_{j}-\bar{x})/\sum_{i}{\left(x_{i}-\bar{x}\right)}^{2})$$ (2)

Where n denotes the total number of spatial units, X_i_ is the observed varicella cases in unit i, $\overset{ˉ}{x}$ is the overall mean, and $w_{\mathrm{ij}}$ stands for the elements of a spatial weight matrix defining the spatial relationship between spatial units. The value of Moran’s I ranges from −1 to 1, where values closer to −1 indicate a stronger tendency toward spatial dispersion, while values closer to 1 suggest a stronger tendency toward spatial clustering of the variable of interest (29). The global Moran test was performed to determine the statistical significance of global spatial autocorrelation. The spatial proximity matrix was generated through the k-nearest neighbors algorithm and the spatial empirical Bayesian approach (30). Specifically, we assume the observed number of varicella cases Y_i_ follows a Poisson distribution with mean E_i_ $\theta_{i}$ , where E_i_ denotes the expected number of cases and $\theta_{i}$ represents the underlying relative risk in spatial unit i. Based on the empirical Bayes framework, the prior distribution of $\theta_{i}$ follows a Gamma distribution, the parameters of which are estimated from the prior mean and variance from the observed risk within a predefined spatial neighbor unit. The smoothed estimate was then calculated as a weighted average of the observed crude risk and the locally estimated prior mean, with weights determined by the relative magnitude of within-area variance and local prior variance (31). Specific spatial cluster patterns (high-high, high-low, low-high, and low-low) were detected through leveraging local spatial autocorrelation analysis by computing local index spatial autocorrelation (LISA), where the high-high pattern stands for spatial units with a high morbidity rate surrounded by regions that also exhibit a high morbidity rate. The Local Moran’s I can be interpreted as a disaggregation of the global statistic, quantifying the contribution of each spatial unit to the overall measure of spatial autocorrelation. We computed LISA statistics using the Equation (3):


$$I_{i}=(x_{i}-\bar{x})\sum_{j}w_{\mathrm{ij}}\;(x_{j}-\bar{x})$$ (3)

All LISA mapping in this study used empirical Bayes-smoothed rates, determined by Equation (4):


$$\hat{R_{i}}=W_{i}\cdot r_{i}+(1-W_{i})\cdot \mu_{i}^{*}$$ (4)

Where $W_{i}$ stands for the spatial Bayesian weight calculated by the variability parameters from the crude rate in each spatial unit, $\mu_{i}^{*}$ represents the average incidence rate in the surrounding spatial context of the unit i . All spatial autocorrelation analysis was based on the smoothing incidence rate computed by the above method. The entire analysis was performed in R (version 4.4.3).

#### Spatial–temporal scan statistics

The spatial–temporal scan statistics proposed by Kulldorff and Nagarwalla (32) along with the rsatscan package (Version 1.0.9) in R were used to detect varicella clusters in both space and time (33). The fundamental principle of scan statistics involves moving a cylinder window dynamically through space and time dimensions, where the circular or elliptical bottom of the cylinder represents geographical spaces, while the height of the cylinder corresponds to the temporal duration of potential clusters (34). The cases observed in each scanning window were compared to the expected counts of cases under the null hypothesis of a random spatiotemporal distribution pattern to generate the relative risk of contracting varicella within the window relative to outside (28). Monte Carlo simulation was used to assess statistical significance, with a *p*-value threshold of 0.05 used to determine significance. The log likelihood ratio (LLR) was used to identify potential clusters, with the cluster exhibiting the maximum LLR considered the most likely cluster.

To assess the influence of spatial window size, we evaluated maximum spatial cluster sizes from 10 to 50% of the population at risk. Based on sensitivity analysis, a spatial window of 30% was selected, as the largest detected cluster encompassed less than 15% of the total geographic units, balancing cluster detectability and spatial specificity. The maximum temporal cluster size was set at 50% of the total study period. Case data were temporally aggregated at the monthly level, with a minimum of five cases in one cluster, consistent with the definition of a cluster from the varicella surveillance scheme of Zhejiang Province.

### Epidemiological characteristics

**Figure 1:**
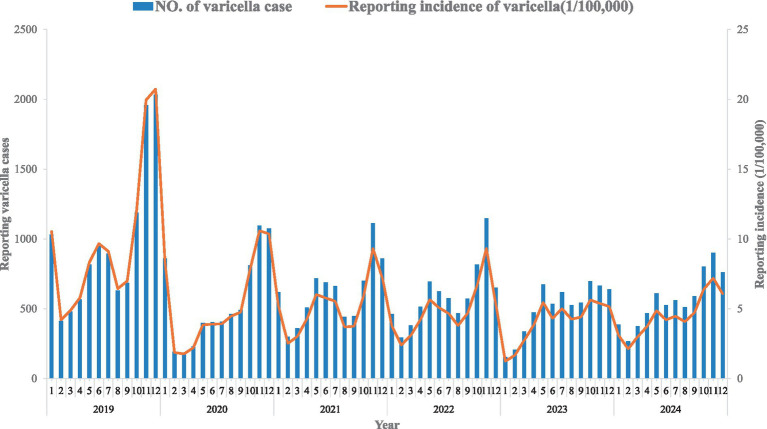
Reported monthly cases and incidence rate of varicella from 2019 to 2024.

**Figure 2:**
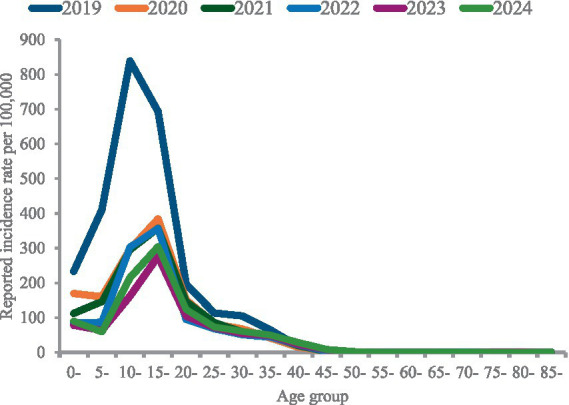
Reported varicella incidence rate by the age group.

Between 2019 and 2024, a total of 44,740 varicella cases were reported in Hangzhou, with an average annual incidence rate of varicella of 60.59/100,000, including 23,900 male cases and 20,840 female cases. Epidemiological characteristics of varicella in Hangzhou from 2019 to 2024 are shown in Figures 1, 2. The temporal tendency of varicella morbidity (Figure 2) illustrates that the incidence rate peaked in 2019 at 97.75 per 100,000, fluctuating between 50 and 60 per 100,000 from 2020 to 2022, while the bottom of the incidence rate was observed in 2023 at 47.4 per 100,000, but it slightly bottoms out in 2024 at 52.23 per 100,000.

**Figure 3:**
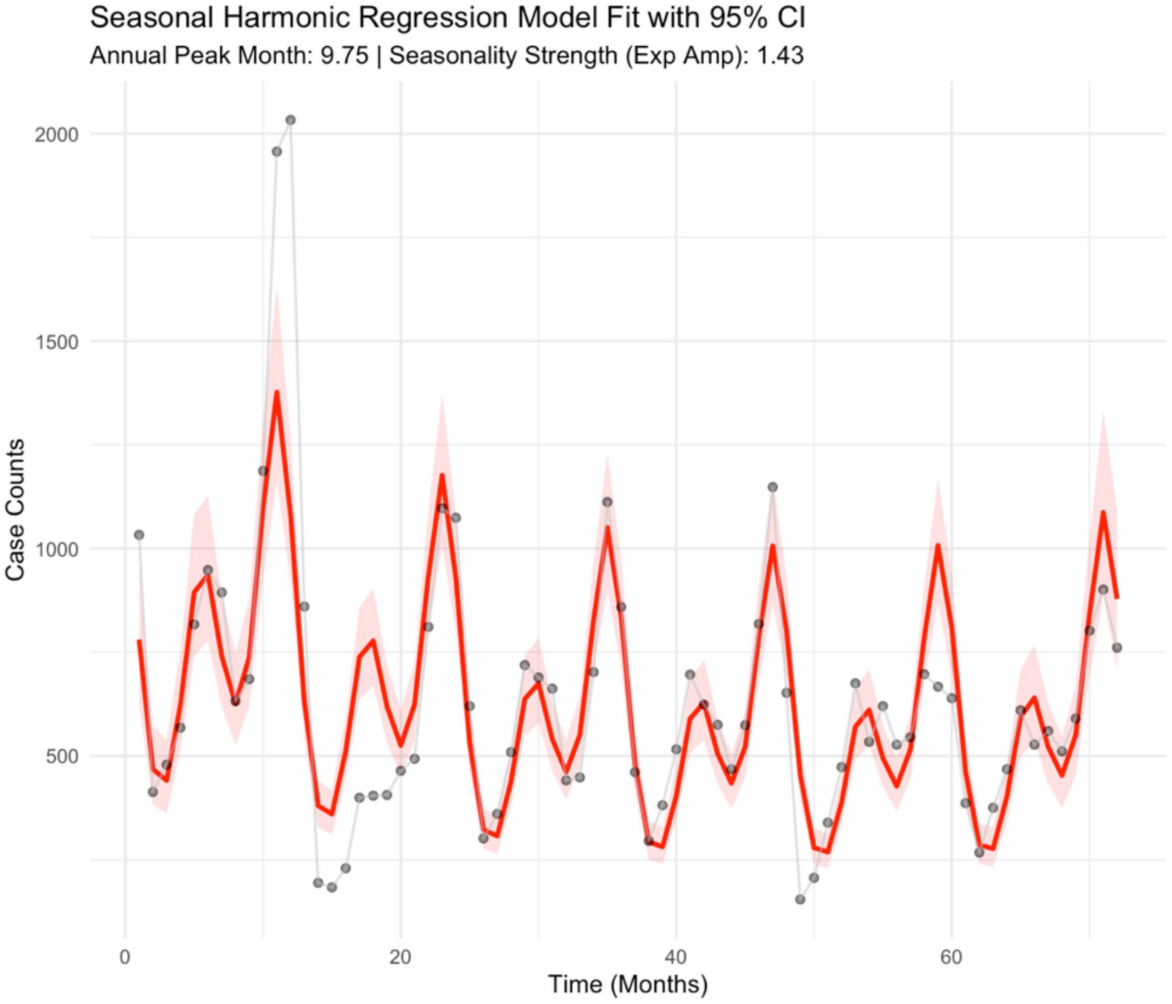
Seasonality quantification with the harmonic regression model.

The exploratory seasonality analysis in the epidemic curve (Figure 1) revealed a bimodal seasonality pattern of varicella incidence in Hangzhou, with the first peak of varicella cases observed from May to July, whereas the second peak typically occurs in the winter months from October of the year to November. Results from harmonic regression analysis provide a more evident and quantitative outcome for the seasonality pattern of varicella incidence in Hangzhou. The regression modeling plot is shown in Figure 3. Specifically, the semi-periodicity contribution ratio (54.8%) from the regression model showed a bimodal pattern of varicella incidence over the study period. The estimated annual seasonality strength was 1.43 (95% CI: 1.294–1.693), indicating that peak incidence was approximately 43% higher than the seasonal mean. The annual amplitude on the log scale was 0.354, reflecting moderate but clearly defined seasonal fluctuation. The timing of the annual peak was estimated to occur at month 9.75, corresponding to late September to early October, with a 95% confidence interval spanning from month 8.946 to 10.334.

**Figure 4:**
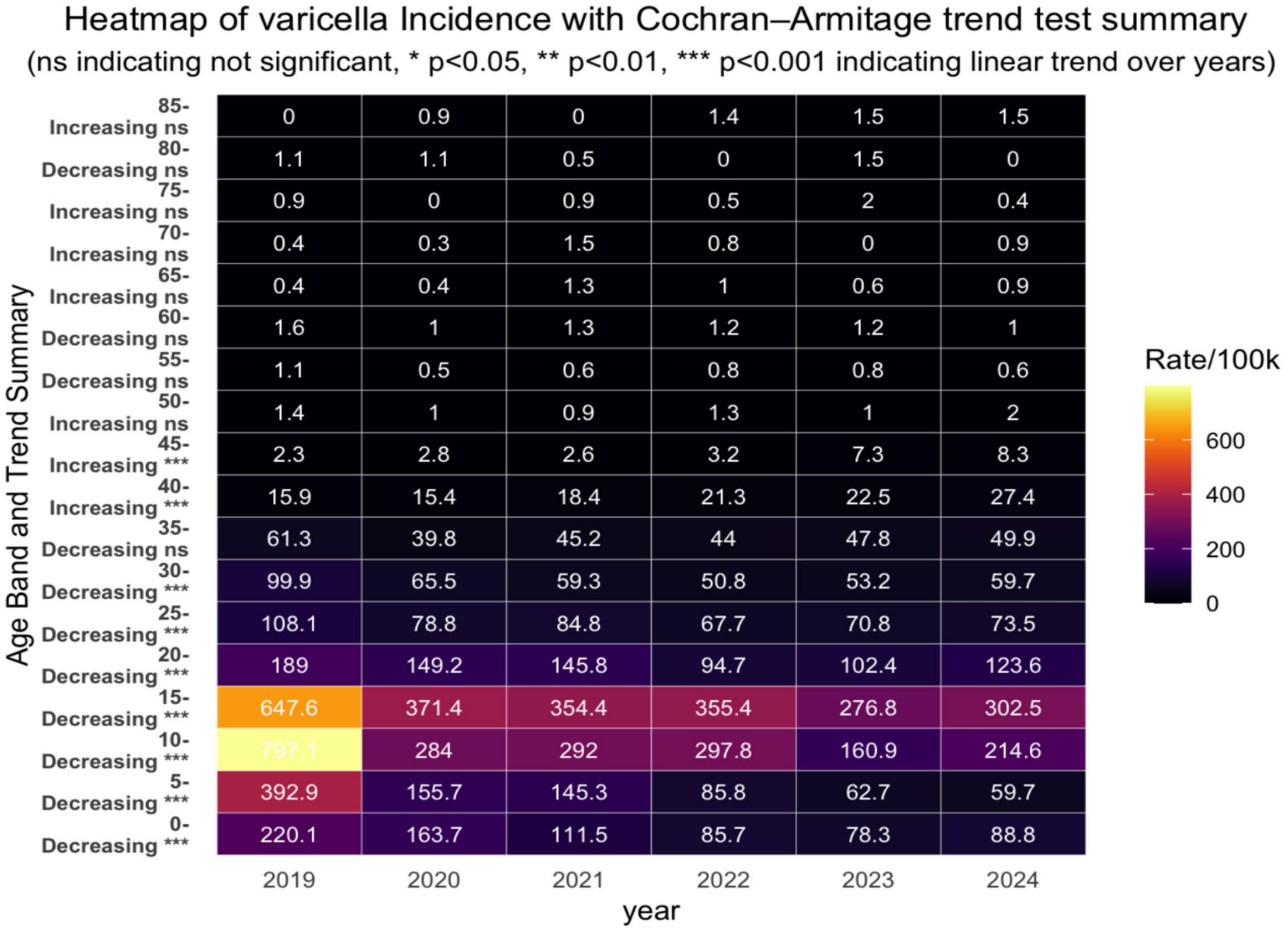
Heatmap of age-band incidence by year with the Cochran-Armitage trend test.

**Figure 5:**
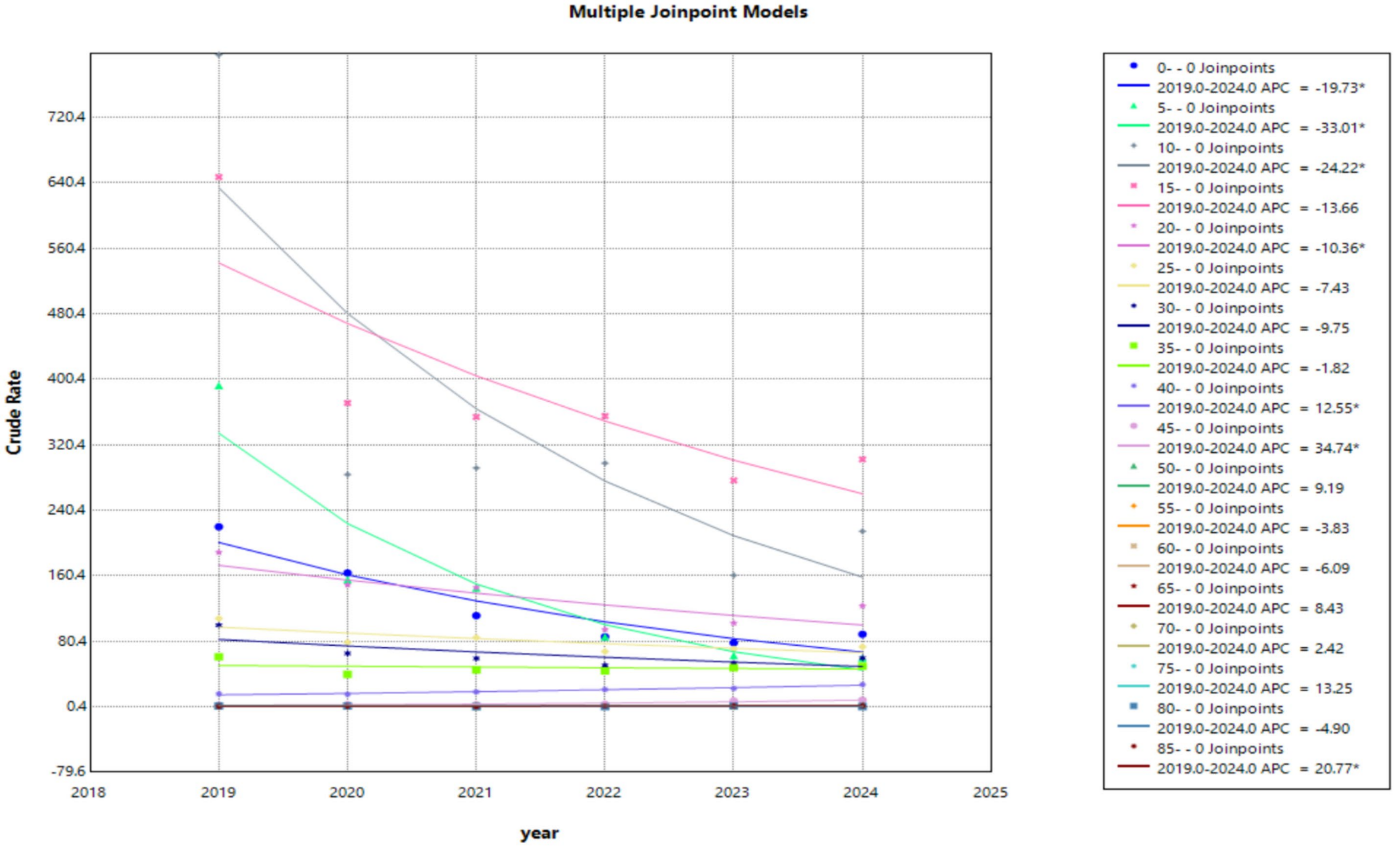
Temporal trend of age-specific incidence of varicella from joinpoint modeling, 2019–2024.

The age-specific incidence distribution by year and results of Cochran–Armitage trend tests within each age group (Figure 4) revealed that varicella incidence in Hangzhou over the study period exhibited a pronounced age gradient, with the highest rates consistently observed among young children, particularly those aged 5–9 and 10–14 years, followed by a sharp decline with increasing age. In 2019, the highest incidence rates were concentrated in the youngest three age groups, indicating that early childhood accounted for the most reported cases at the beginning of the study period. From 2020 onward, however, the age distribution of incidence changed markedly; the youngest age groups experienced substantial and statistically significant declines (*p* < 0.001), while children aged 10–14 and 15–19 years gradually emerged as the dominant incidence groups. This transition reflects both the pronounced reduction in varicella incidence among young children and the relatively slower decline in older pediatric and adolescent groups. This shift is also consistent with results from our joint-point regression modeling, as shown in Figure 5, revealing that children in the younger age band (5–10 years old) experienced a greater incidence decline (APC: 33.01) than those in the 10–19 age band (APC10-14: 24.22, APC 15–19: 13.66). These findings highlight a marked temporal decline in varicella burden concentrated in younger populations, alongside relatively stable and low incidence in older age groups, underscoring the strong age-specific epidemiological pattern of varicella during the study period.

### Spatial pattern

**Figure 6:**
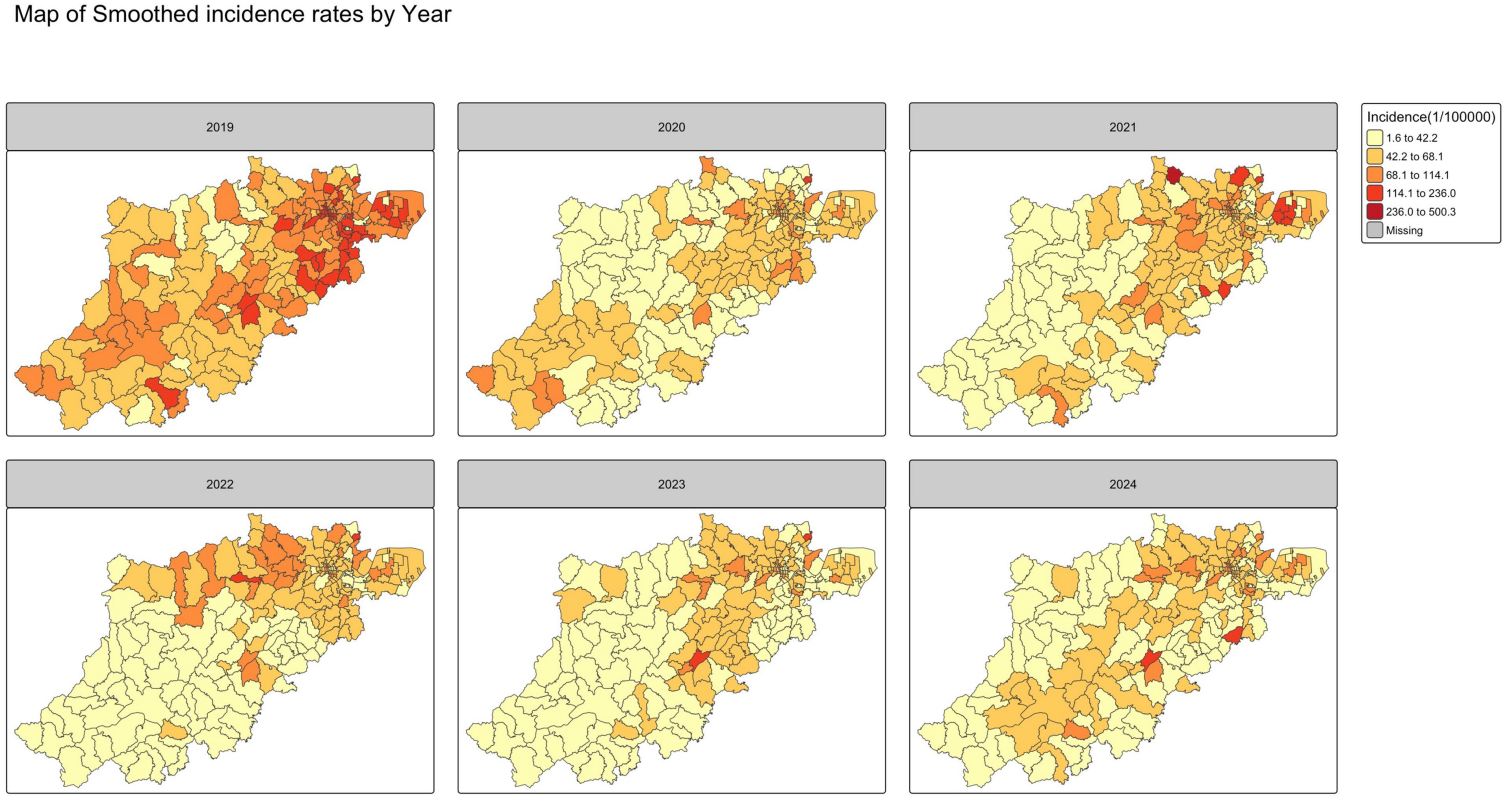
Average smoothed incidence rate mapping by calendar year.

**Figure 7:**
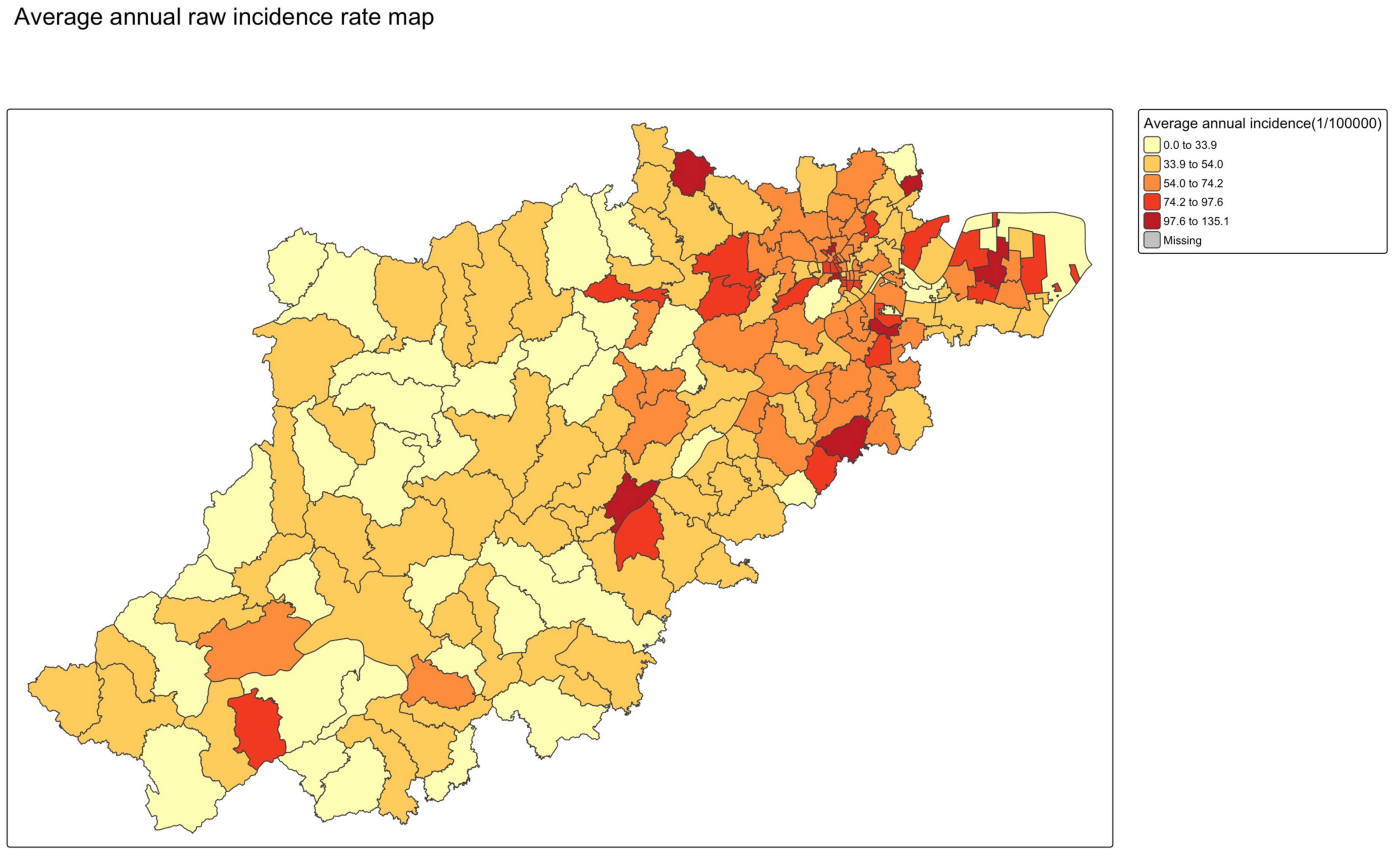
Average annual raw incidence rate mapping.

**Figure 8:**
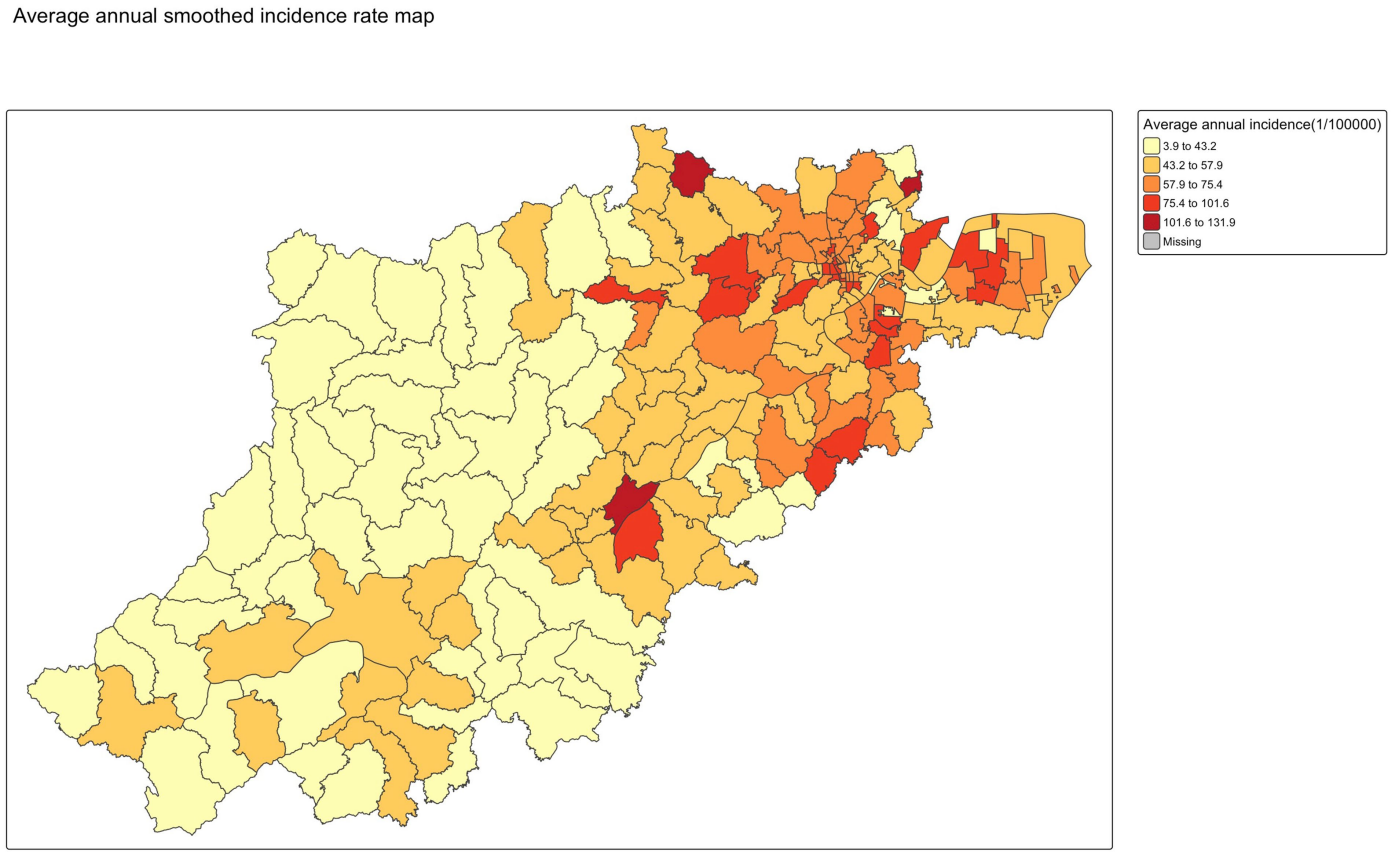
Average annual smoothed incidence rate mapping.

The smoothed annual incidence of varicella map from 2019 to 2024 in Figure 6 revealed a consistent spatial distribution pattern across the years, suggesting that varicella incidence was generally higher in central urban districts and several suburban areas in northeast compared to other peripheral regions except for 2019. Notably, the overall morbidity rate in 2019 was higher than in the subsequent 5 years, suggesting a temporal decline in varicella incidence. No significant differences were observed between the smoothed rates using the SEB method and the raw incidence rates, as shown in Figures 7, 8.

### Spatial autocorrelation

**Figure 9:**
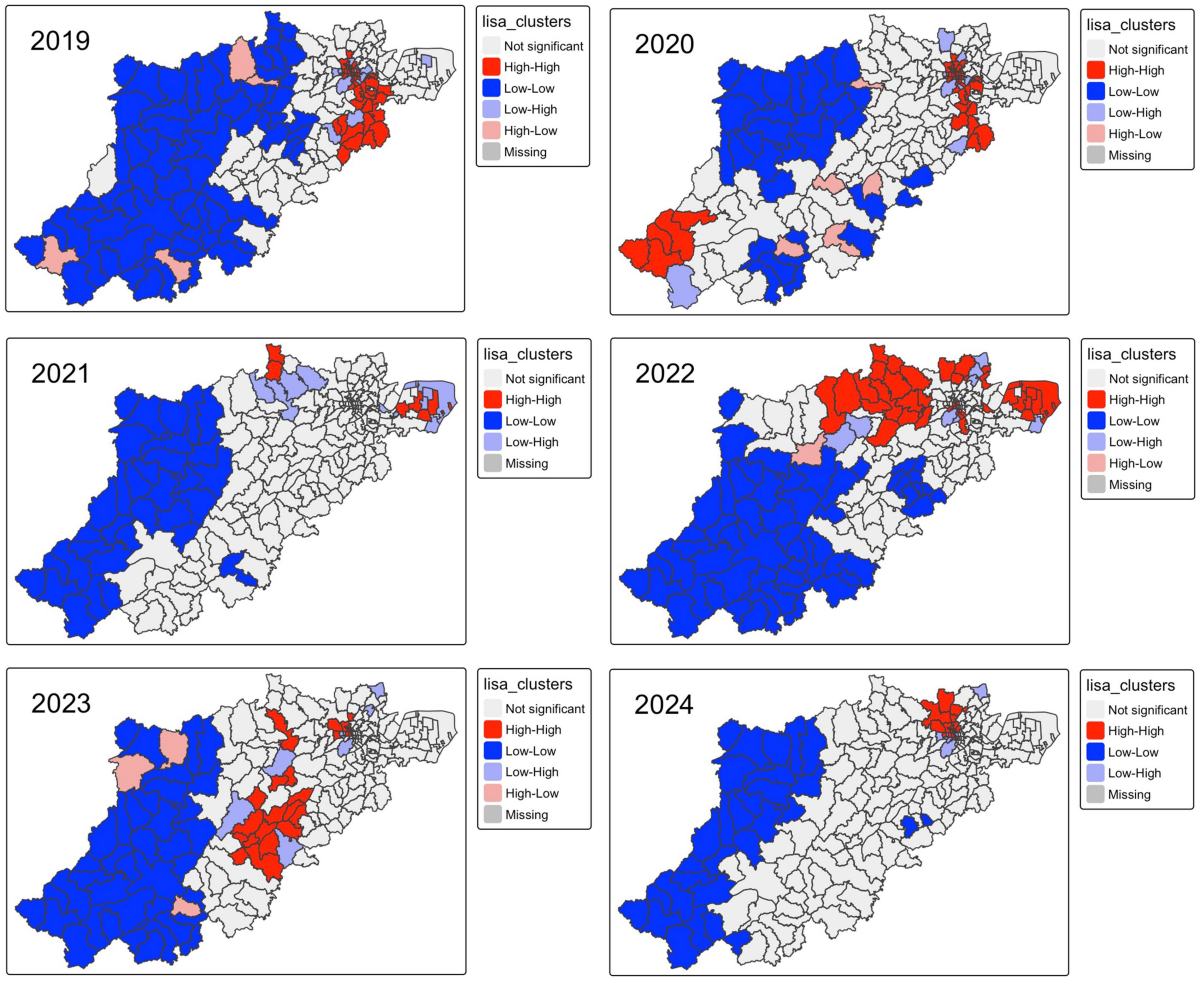
Varicella LISA clusters, 2019–2024.

The results of the global autocorrelation test in Table 1 show a statistically significant positive spatial autocorrelation for varicella morbidity across townships in Hangzhou from 2019 to 2024. Similar results were also observed when the incidence rate was averaged for 6 years at township levels (*p-*value 0.05), indicating that the spatial distribution pattern of varicella in Hangzhou at township levels was non-random. Specifically, the Local Moran clusters map of varicella incidence revealed that the high-high clusters were mainly concentrated in downtown townships and several suburban towns near the city center in 2019 and 2020, while five LISA clusters were identified in Chunan County in 2020 (Zhongzhou town, Fenkou town, Langchuan town, Jiangjia town, and Zitong town), as shown in Figure 4. High-high clusters had significantly decreased in 2021, and the LISA cluster pattern had shifted from urban areas concentrated to predominantly located in peripheral areas, as only six hotspots of varicella were recognized by LISA analysis in 2021 (Bai Zhang Town & Lu Niao Town in Yuhang district, Dangwan Town & Nan Yang Street in Xiaoshan district, Yipeng Town & Linjiang Town in Qiantang district). However, in 2022, 38 LISA clusters were detected across 9 districts (Linan, Linpin, Xihu, Gongshu, Shangcheng, Xiaoshan, and Qiantang), with a predominant concentration (71%) in suburban townships, including 27 in peripheral areas and 11 in urban townships. Hotspots slightly declined in 2023 and 2024, with 28 and 19 township-level clusters detected, respectively, in 5 districts (Figure 9).

**Table 1:** Results of the global Moran test.

Year	Moran I	Expectation	Variance	Statistic	*p*-value
2019	0.706374859	−0.005181347	0.001817847	16.6890109	7.878E-63
2020	0.404993617	−0.005181347	0.001809995	9.641185438	2.6783E-22
2021	0.095276993	−0.005181347	0.001307046	2.778693017	0.0027289
2022	0.323192324	−0.005181347	0.001814662	7.708509464	6.3648E-15
2023	0.284099559	−0.005181347	0.001766101	6.883542144	2.9191E-12
2024	0.400626661	−0.005181347	0.001770559	9.644181905	2.6012E-22
Average	0.443151546	−0.005181347	0.001718609	10.81463883	1.4672E-27

### Spatiotemporal cluster analysis

**Figure 10:**
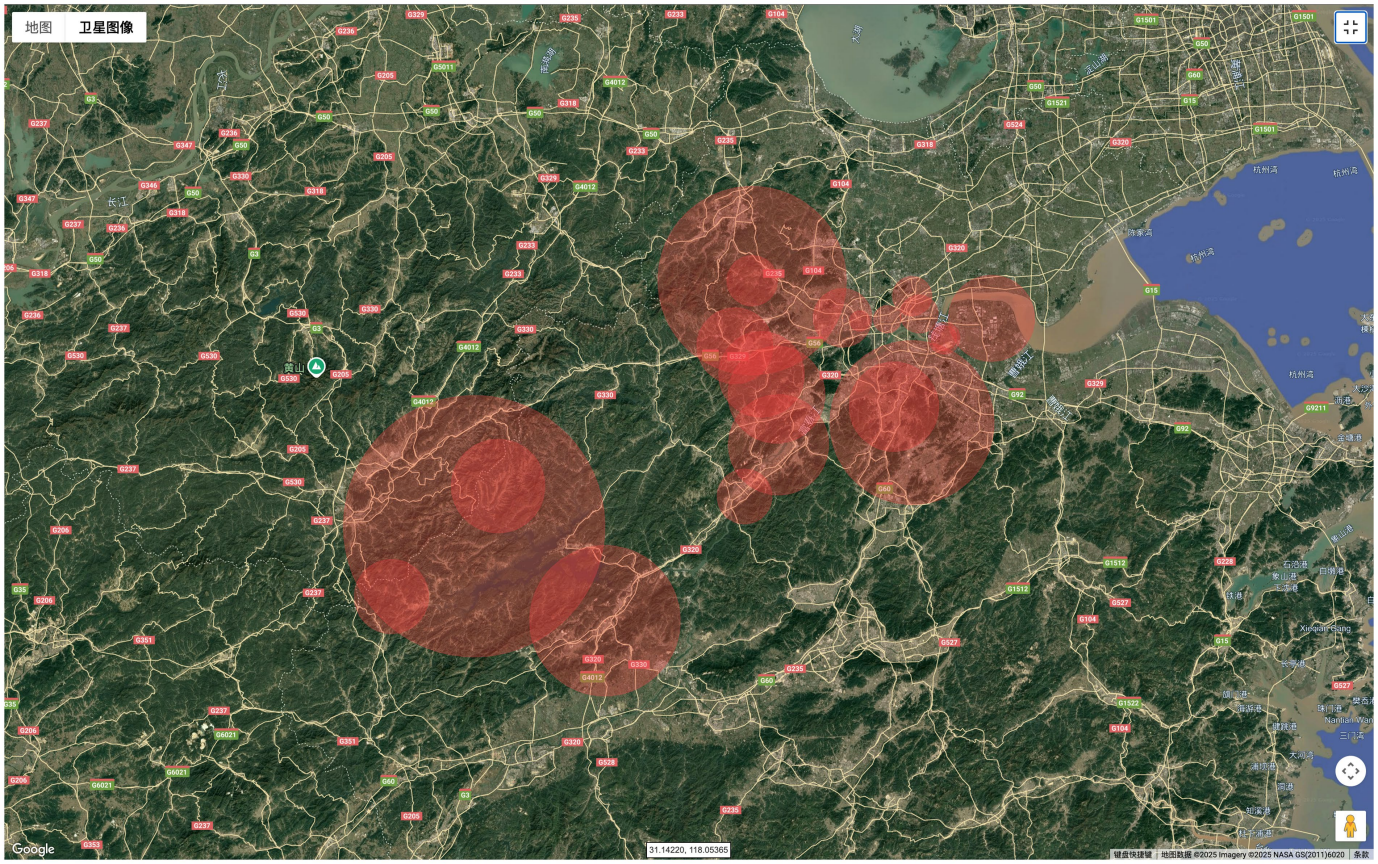
Cluster mapping detected by space–time scan statistics from 2019 to 2024 in Hangzhou.

The retrospective spatiotemporal scan analysis identified a total of 34 statistically significant varicella clusters in Hangzhou from 2019 to 2024 (*p* < 0.05), indicating a notable spatiotemporal aggregation of varicella cases during the study period, as illustrated in Figure 10. Interestingly, only 3 clusters were identified in the downtown area (cluster 23, cluster 24, and cluster 33) that have been highlighted in bold in Appendix Table 2, whereas all remaining clusters were in suburban regions. The most likely cluster occurred in Huanghu Town, Yuhang District, comprising 83 cases between 1 September and 30 November 2021. This cluster exhibited a relative risk (RR) of 13.01, suggesting that individuals within the specified spatial and temporal window were 13.01 times more likely to contract varicella compared to those outside the cluster (log-likelihood ratio [LLR] = 136.38, *p* < 0.05). The secondary cluster encompassed 30 contiguous townships/streets across four districts—Shangcheng, Xiaoshan, Binjiang, and Fuyang—located in the southern part of Hangzhou. This cluster spanned the entire year of 2019 (1st January to 31st December), with a relative risk of 1.32 and an LLR of 127.88 (*p* < 0.05). Detailed information regarding the remaining 36 clusters is provided in Appendix Table 2.

## Discussion

This is the first study that investigates epidemiological characteristics and reveals the spatiotemporal pattern of varicella at township level since varicella has been mandatory reported to CIDCP since 2019 in Hangzhou. Temporally, our study observed a continuous decline in the reported incidence of varicella in Hangzhou from 2019 to 2024. This trend is especially striking from 2019 (97.75 per 100,000) to 2020 (53.76 per 100,000), which could be ascribed to the non-pharmaceutical intervention undertaken during the COVID-19 pandemic and the increasing coverage of varicella vaccination in Hangzhou (35, 36). This finding was consistent with the temporal trend of varicella reported elsewhere in China (37, 38).

Furthermore, it is worthwhile to note that our study identified a bimodal peak distribution of varicella cases in Hangzhou, which is consistent with results reported by previous studies (39–41). One plausible explanation underlying this phenomenon has been demonstrated by Suzuki et al. (41) and Xu et al. (39), indicating that the existence of preferable metrological temperatures for varicella dynamics and the school terms when the most susceptible population gathers promotes transmission of varicella in spring and winter seasons. These results are similar to findings from previous scholars (38, 42). This current study also found that a dominant age group transition of varicella incidence from the 5–9-year-old to 10–19-year-old age group from 2019 to 2024. Cases in 5–9 age group have declined from 1,394 cases in 2019 to 461 in 2024, whereas both the 10–15 age group and the 15–19 age group have observed a slight increase in varicella cases since 2020. The position paper from WHO for the varicella and herpes zoster vaccine in 2014 argued that when the varicella vaccine coverage remains under 80% for a long duration, although a decrease in overall counts of cases could be observed, it is expected that a shift of varicella cases to an older age group of children (43). It is therefore suggested that future research could be targeted at varicella vaccination coverage and its spatial–temporal distribution, as well as the relationship between varicella vaccine uptake and the incidence of varicella outbreak, to ensure the target population has already constructed sufficient immune barriers (13).

Global autocorrelation in our study confirmed the existence of a positive aggregation pattern of varicella at township level in Hangzhou in every single year from 2019 to 2024. However, insights from our LISA analysis, along with retrospective spatial cluster analysis, have challenged the conventional notion that infectious respiratory disease clusters are prone to occur in densely populated areas with a higher socioeconomic index. Our findings revealed that varicella clusters tend to happen in the relatively underdeveloped regions in Hangzhou, although the cluster area has evolved. However, our findings are supported by evidence from Li et al. (15) and Lee et al. (44), who argued that the socioeconomic status played a pivotal role in the varicella cluster spatial pattern. Specifically, they found that a higher per capita GDP was related to reduced varicella transmission risk and several other social determinants, including per capita residential building area, percentage of rental housing, and spatial features of chickenpox outbreaks. On the other hand, although the varicella vaccine is the most effective way to mitigate varicella transmission, a plethora of evidence has identified an increasing number of breakthrough varicella cases with a one-dose varicella vaccination history; a varicella outbreak analysis by Wang et al. found that 96.6% of breakthrough cases had a history of one-dose vaccination (11), revealing the limited protective effect of a one-dose schedule for varicella. However, previous studies on varicella vaccination coverage in Hangzhou have found that the two-dose coverage rate was lower in suburban areas (52.48%) compared to downtown areas (62.5%) (35). As a matter of fact, some researchers have provided solutions to tackle inequality in vaccine accessibility between different socioeconomic groups. Hu et al. (45) suggested the adoption of 2-dose varicella vaccination as one of the school-entry vaccination requirements. Other researchers have also put forward various recommendations to improve the accessibility of the varicella vaccine, such as incorporating the varicella vaccine into the National Immunization Program (NIP) vaccines (46) and replacing the MMR vaccines with the MMRV vaccine^2 11^. Therefore, the effectiveness and cost-effectiveness of the MMRV vaccine schedule and incorporating the MMRV vaccine into NIP in comparison with the present self-paid varicella vaccine strategy in Hangzhou warrant further research.

## Limitations and conclusion

Some challenges needed to be noted. First, to explore the township-level spatiotemporal cluster pattern of varicella, cases without an identifiable residential address were excluded from analysis in this study, which may lead to a slight underestimate of varicella morbidity, but this proportion was small (less than 0.5%) and thus will not substantially affect the spatiotemporal pattern and transmission dynamic characteristics of varicella. Second, since varicella has been a legally notifiable disease in Hangzhou since 2019, data were only available for a short duration of 5 years, which hindered us from exploring periodic trends and long-term epidemic characteristics of varicella in Hangzhou. Third, due to the limited socioeconomic data provided by CISDCP surveillance, we were unable to further examine the influence of socioeconomic factors on varicella onset or on the uptake of varicella vaccination. Finally, unlike irregularly shaped administrative boundaries in the real world, spatial scan statistics use a circular or elliptical scanning window to detect the most likely cluster. It may be criticized that several adjacent spatial units without elevated risk are incorporated in the most likely cluster (47). However, unlike undertaking spatial scan statistics at the county-level, township-level analysis diminishes this effect to some extent, since in real-world infectious disease intervention practices, the cost of mistakenly categorizing a county as a high-risk area without actual elevated risk is typically greater than that of misclassifying a township.

In conclusion, this study demonstrates a downward trend in the incidence rate of varicella in Hangzhou over the past 5 years. The reason underlying this phenomenon could be the high varicella coverage rate in Hangzhou. However, the morbidity rate of varicella in young children between 10 and 19 years old has gradually increased, suggesting a potential gap between the current two-dose varicella vaccination coverage in Hangzhou and the threshold required to establish herd immunity barriers, as recommended by the WHO position paper (43). This highlights the need for policymakers and public health professionals to consider disparities in access to non-NIP vaccines when formulating future varicella prevention strategies.

## Data Availability

The original contributions presented in the study are included in the article/Supplementary material, further inquiries can be directed to the corresponding author.
