# Supplementary material for: Spatial–temporal patterns, seasonality, and age-specific trends of varicella in Hangzhou, China, 2019–2024
Source: Front Public Health. 2026 Jan 30;14:1701894. doi: 10.3389/fpubh.2026.1701894 (PMC12901367; doi:10.3389/fpubh.2026.1701894)
Supplement: Supplementary file 1 [file Data_Sheet_1.docx]

**Appendix**

Table 2. LISA clusters of varicella in Hangzhou from 2019 to 2024

| **Year** | **Town/streets (n)** | **Hot-spot lists** |
| --- | --- | --- |
| 2019 | 45 | Kangqiao Street, Xiangfu Street, Hemu Street, Gongchenqiao Street, Xiaohé Street, Gudang Street, Cuiyuan Street, Xixi Street, Mishixiang Street, Hushu Street, Daguan Street, Dongxin Street, Lingyin Street, Beishan Street, Wulin Street, TianShui Street, Zhaohui Street, Changqing Street, Chaoming Street, Kaixuan Street, Zhanongkou Street, Hubin Street, Xiaoying Street, Caihe Street, Qingbo Street, Nanxing Street, Ziyang Street, Changhe Street, Xixing Street, Ningwei Street, Beigan Street, Xinjie Street, Wenyan Street, Chengxiang Street, Xintang Street, Shushan Street, Suoqian Town, Linpu Town, Daicun Town, Yushan Township, Lishan Town, Jinhua Town, Puyang Town, Heshang Town, Louta Town. |
| 2020 | 45 | Fen kou Town, Zhong zhou Town, Lang chuan Township, Jiang jia Town, Zi tong Town, Jinhua Town, Pu yang Town, Lin pu Town, Yi qiao Town, Shu shan Street, Wen yan Street, Chang he Street, Xi xing Street, Ning wei Street, Nan xing Street, Wang jiang Street, Hu bin Street, Xiao ying Street, Qing bo Street, Wu lin Street, Tian shui Street, Chang qing Street, Chao ming Street, Kai xuan Street, Ling yin Street, Bei shan Street, GudangStreet, Cui yuan Street, Xi xi Street, Mi shi xiang Street, Zhao hui Street, Wen hui Street, Zha nong kou Street, Xiao he Street, Hu shu Street, Xiang fu Street, Shang tang Street, Da guan Street, Dong xin Street, Shi qiao Street, He mu Street, Gong chen qiao Street, Kang qiao Street. |
| 2021 | 6 | Bai Zhang Town, Lu Niao Town, Nan Yang Street, Yi Peng Street, Dangwan Town, Linjiang Street. |
| 2022 | 38 | Tianmu Mountain Town, Taihu Source Town, Gaohong Town, Jinbei Street, Banqiao Town, Qingshan Lake Street, Lu Niao Town, Bai Zhang Town, Huanghu Town, Pingyao Town, Zhongtai Street, Xianlin Street, Yuhang Street, Cangqian Street, Jingshan Town, Changhe Street, Ziyang Street, Wangjiang Street, Xiaoying Street, Qingbo Street, Hubin Street, Lingyin Street, Beishan Street, Wulin Street, Xixi Street, Wenhui Street, Weikeng District, Nanyuan Street, Donghu Street, Tangxi Town, Chongxian Street, Renhe Street, Dangwan Town, Linjiang Street, Xinwan Street, Qianjin Street, Yipeng Street, Hezhuang Street. |
| 2023 | 28 | Kangqiao Street, Cuiyuan Street, Xixi Street, Mishixiang Street, Sandun Street, Xiangfu Street, Xiaohé Street, Hemu Street, Jiangcun Street, Wulin Street, Hushu Street, Gongchenqiao Street, Qingshan Lake Street, Yongchang Town, Chunjiang Township, Xukou Town, Hengcun Town, Eshan Shezu Township, Qintang Township, Jiuxian Street, Fuchunjiang Town, Tongjun Street, LuZhu Town, Chengnan Street, Changkou Town, Jiangnan Town, Xintong Township, Lushan Street. |
| 2024 | 19 | Renhe Street, Chongxian Street, Liangzhu Street, Sandun Town, Xiangfu Street, Shangtang Street, Hemu Street, Gongchenqiao Street, Xiaohé Street, Wenxin Street, Cuiyuan Street, Dongxin Street, Daguan Street, Hushu Street, Xixi Street, Mishixiang Street, Beishan Street, Wulin Street, TianShui Street. |

Table 3. 34 clusters detected by the space-time scan statistics on varicella morbidity in Hangzhou, China, 2019-2024

| Administrative Division Codes of Zhejiang Province, China | Coordinates / radius.. | Span | Time frame | Number of cases. | Expected cases | Observed / expected.. | Test statistic | P-value |
| --- | --- | --- | --- | --- | --- | --- | --- | --- |
| 330110113 | (30.468702 N, 119.818581 E) / 0 km | 0 km | 2021/9/1 to 2021/11/30 | 83 | 6.38 | **13.01** | 136.381763 | < 0.00000000000000001 |
| 330109104, 330109103, 330109105, 330109107, 330109101, 330109102, 330109106, 330109003, 330109004, 330109012, 330109403, 330109108, 330109001, 330111207, 330109002, 330109100, 330109014, 330111108, 330108002, 330106110, 330108001, 330111118, 330108003, 330109113, 330109013, 330109503, 330102008, 330111117, 330102009, 330111005 | (29.998093 N, 120.305380 E) / 28.42 km | 50.53 km | 2019/1/1 to 2019/12/31 | 3272 | 2484.17 | 1.32 | 120.873743 | < 0.00000000000000001 |
| 330109006, 330109503, 330109401, 330109005 | (30.261032 N, 120.420685 E) / 5.52 km | 10.40 km | 2021/6/1 to 2021/7/31 | 102 | 17.90 | 5.70 | 93.463023 | < 0.00000000000000001 |
| 330113100 | (30.463131 N, 120.198209 E) / 0 km | 0 km | 2021/11/1 to 2022/1/31 | 132 | 35.35 | **3.73** | 77.374805 | < 0.00000000000000001 |
| 330110111, 330110112, 330110113, 330112102, 330110109, 330112008, 330112007, 330110110, 330112103, 330112006, 330110013, 330110012, 330110014, 330112005, 330112002, 330110010, 330112116, 330112115 | (30.439436 N, 119.730740 E) / 32.50 km | 56.16 km | 2022/6/1 to 2022/12/31 | 834 | 547.39 | 1.52 | 65.493092 | < 0.00000000000000001 |
| 330111200, 330111110, 330111004, 330111103, 330122113, 330111204, 330111111, 330111112, 330111115, 330111001, 330111002, 330111105, 330111201, 330111206, 330122003 | (29.929261 N, 119.825580 E) / 17.43 km | 32.64 km | 2023/1/1 to 2024/7/31 | 638 | 448.98 | 1.42 | 35.553325 | 0.0000000000012 |
| 330112115, 330112005, 330111206, 330111105, 330112002, 330112006, 330112007, 330111006, 330110014 | (30.148393 N, 119.764294 E) / 14.86 km | 27.95 km | 2021/10/1 to 2021/11/30 | 160 | 79.79 | 2.01 | 31.191184 | 0.00000000011 |
| 330109101 | (29.951851 N, 120.168191 E) / 0 km | 0 km | 2024/9/1 to 2024/10/31 | 33 | 5.65 | **5.84** | 30.916341 | 0.00000000015 |
| 330127109, 330127108 | (29.455133 N, 118.434458 E) / 12.98 km | 12.98 km | 2019/1/1 to 2019/1/31 | 21 | 2.36 | **8.89** | 27.250092 | 0.0000000068 |
| 330109102 | (30.000520 N, 120.158093 E) / 0 km | 0 km | 2020/12/1 to 2020/12/31 | 23 | 3.15 | **7.30** | 25.865731 | 0.000000029 |
| 330113002, 330113005, 330113004, 330113003, 330114001, 330113001 | (30.391725 N, 120.306049 E) / 6.84 km | 13.24 km | 2022/2/1 to 2024/3/31 | 1343 | 1102.52 | 1.22 | 25.168514 | 0.000000060 |
| 330109100 | (29.895639 N, 120.111083 E) / 0 km | 0 km | 2021/8/1 to 2021/10/31 | 25 | 4.00 | **6.25** | 24.828975 | 0.000000085 |
| 330111201 | (29.887758 N, 119.995913 E) / 0 km | 0 km | 2021/5/1 to 2021/5/31 | 10 | 0.34 | **29.80** | 24.280683 | 0.00000015 |
| 330109106 | (30.065255 N, 120.172698 E) / 0 km | 0 km | 2021/5/1 to 2021/6/30 | 38 | 9.55 | 3.98 | 24.048552 | 0.00000019 |
| 330112103 | (30.329513 N, 119.561365 E) / 0 km | 0 km | 2019/11/1 to 2019/11/30 | 19 | 2.22 | 8.55 | 24.006541 | 0.00000020 |
| 330127211, 330127107, 330127210, 330127104, 330127214, 330127106, 330127201, 330127212, 330127208, 330127203, 330127200, 330127108, 330127110, 330127216, 330127100, 330127205, 330127109, 330127102, 330127111, 330127103, 330127206 | (29.674495 N, 118.731430 E) / 45.43 km | 83.55 km | 2020/2/1 to 2020/10/31 | 119 | 60.78 | 1.96 | 21.774268 | 0.0000021 |
| 330114001, 330114002, 330113005, 330113002 | (30.336113 N, 120.330997 E) / 6.63 km | 11.67 km | 2024/11/1 to 2024/12/31 | 200 | 120.84 | 1.66 | 21.677718 | 0.0000023 |
| 330182109 | (29.379182 N, 119.201648 E) / 0 km | 0 km | 2019/11/1 to 2019/11/30 | 26 | 5.08 | **5.12** | 21.525365 | 0.0000027 |
| 330112006, 330112007, 330112002, 330112005, 330112008, 330112102 | (30.237316 N, 119.668754 E) / 13.19 km | 20.23 km | 2023/10/1 to 2024/1/31 | 149 | 83.41 | 1.79 | 20.901830 | 0.0000051 |
| 330112109 | (30.096919 N, 119.170912 E) / 0 km | 0 km | 2019/6/1 to 2019/6/30 | 9 | 0.39 | **22.96** | 19.595348 | 0.000020 |
| 330182110 | (29.322081 N, 119.138930 E) / 0 km | 0 km | 2021/11/1 to 2021/11/30 | 14 | 1.61 | **8.72** | 17.926801 | 0.00011 |
| 330106109 | (30.324334 N, 120.055943 E) / 0 km | 0 km | 2020/10/1 to 2021/1/31 | 146 | 85.21 | 1.71 | 17.874140 | 0.00012 |
| **330102016, 330105019, 330102015, 330102013, 330105018, 330105017** | **(30.319841 N, 120.216478 E) / 4.79 km** | **6.83 km** | **2020/3/1 to 2020/3/31** | **31** | **8.58** | **3.61** | **17.394345** | **0.00020** |
| **330106109, 330106011, 330105009, 330106013, 330105004, 330110005, 330110010, 330106007, 330105005, 330105003, 330106005, 330110012, 330105002, 330106004, 330106012, 330105008, 330105010, 330105007, 330105001, 330106009** | **(30.324334 N, 120.055943 E) / 10.17 km** | **17.09 km** | **2023/12/1 to 2024/5/31** | **652** | **514.90** | **1.27** | **17.031855** | **0.00029** |
| 330109105, 330109107, 330109106, 330109003, 330109104, 330109103, 330109102, 330109012, 330109101, 330111207, 330109001, 330109004, 330109403, 330108002 | (30.047853 N, 120.240598 E) / 15.54 km | 26.81 km | 2020/4/1 to 2020/9/30 | 332 | 238.61 | 1.39 | 16.371067 | 0.00058 |
| 330182109, 330182003, 330182110, 330182111, 330182001, 330127102, 330182112, 330182002, 330182113, 330127200, 330182101 | (29.379182 N, 119.201648 E) / 26.25 km | 42.92 km | 2021/6/1 to 2021/9/30 | 81 | 40.30 | 2.01 | 15.860426 | 0.00098 |
| 330127104, 330127210, 330127208, 330127211 | (29.800540 N, 118.817609 E) / 16.29 km | 28.97 km | 2019/9/1 to 2019/10/31 | 16 | 2.61 | **6.13** | 15.627421 | 0.0013 |
| 330110111, 330110112 | (30.439436 N, 119.730740 E) / 8.75 km | 8.75 km | 2020/5/1 to 2020/6/30 | 10 | 0.84 | **11.86** | 15.574715 | 0.0013 |
| 330109006, 330109503 | (30.261032 N, 120.420685 E) / 4.46 km | 4.46 km | 2022/1/1 to 2022/5/31 | 45 | 17.18 | 2.62 | 15.523130 | 0.0014 |
| 330111206, 330111105, 330111001, 330112115, 330111006, 330111004,  330111115, 330112005, 330111002, 330110014 | (30.085340 N, 119.818285 E) / 16.83 km | 30.52 km | 2020/11/1 to 2021/2/28 | 225 | 153.43 | 1.47 | 14.628958 | 0.0036 |
| 330122003 | (29.835413 N, 119.680611 E) / 0 km | 0 km | 2019/10/1 to 2019/11/30 | 50 | 21.39 | 2.34 | 13.849182 | 0.0080 |
| 330109501, 330114007, 330114006, 330114005, 330114004, 330109120, 330114003, 330109005 | (30.322413 N, 120.591245 E) / 14.92 km | 15.05 km | 2022/7/1 to 2022/7/31 | 52 | 22.96 | 2.26 | 13.478959 | 0.010 |
| **330105004, 330105009, 330105003, 330105005, 330105002, 330106005, 330105007** | **(30.314572 N, 120.117898 E) / 3.58 km** | **4.63 km** | **2024/11/1 to 2024/11/30** | **86** | **46.48** | **1.85** | 13.413659 | 0.010 |
| 330122004, 330122003, 330122005, 330122101, 330122002 | (29.767941 N, 119.703633 E) / 9.57 km | 18.99 km | 2022/3/1 to 2022/3/31 | 30 | 10.09 | 2.97 | 12.783417 | 0.021 |

**
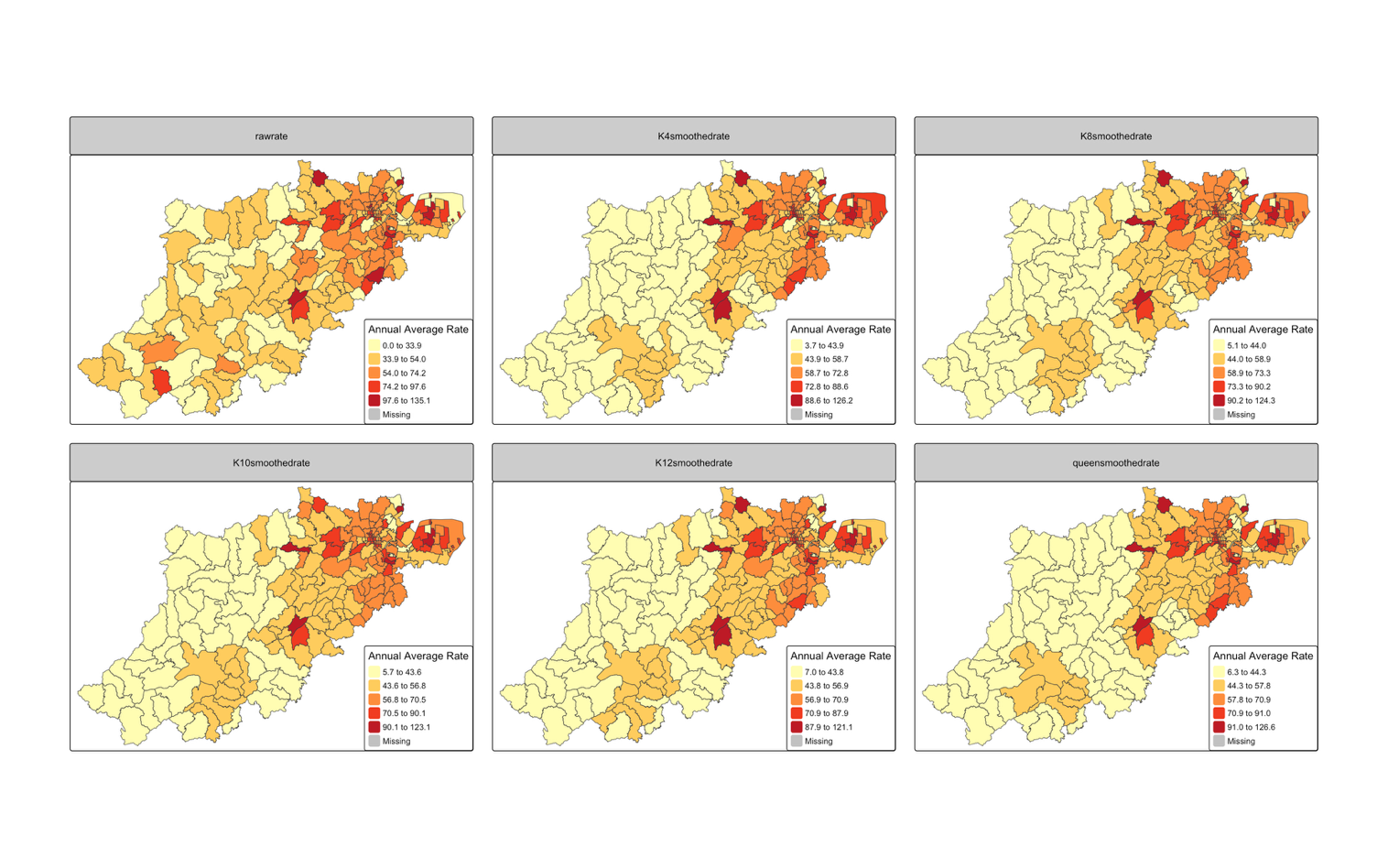
**

**Figure 11. Annual average incidence rate mapping with different spatial weights (k=4, 8, 10, 12; queen weight & raw rate)**

**
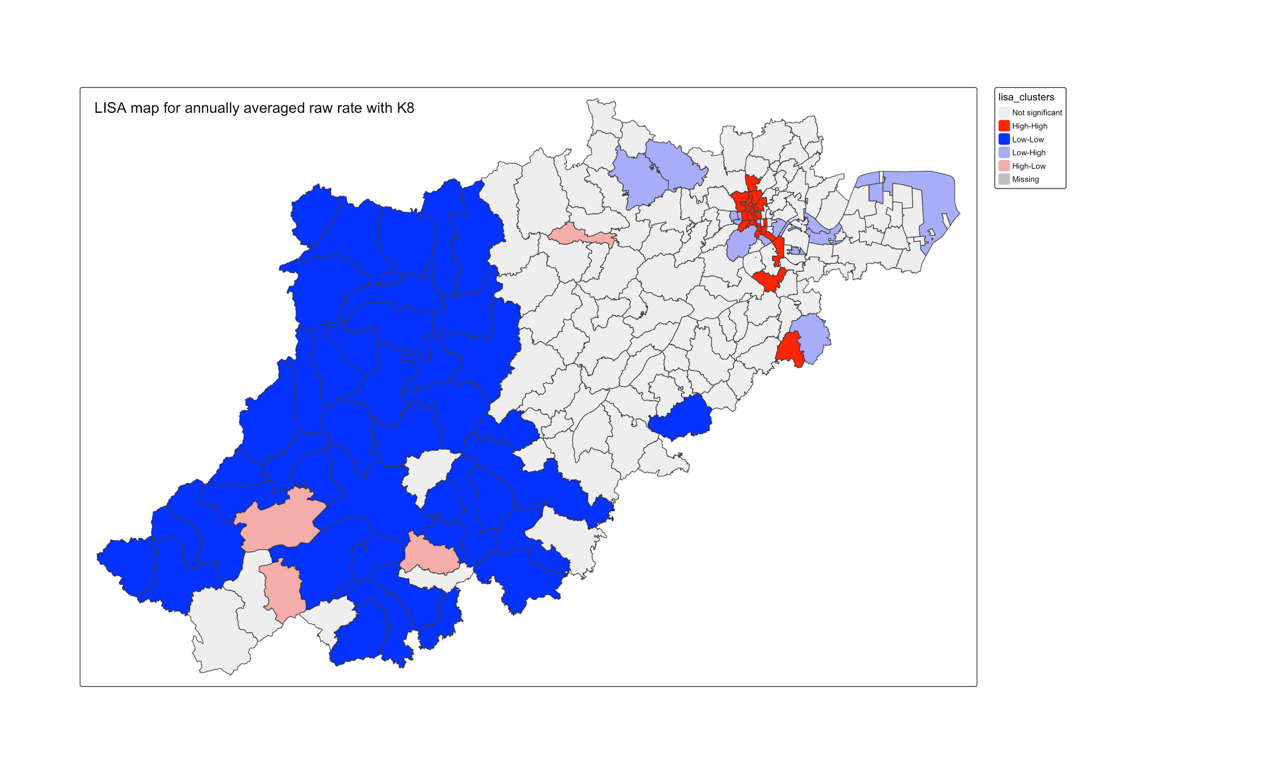
**

**Figure 12. LISA map for annually averaged rate with K = 8**

**
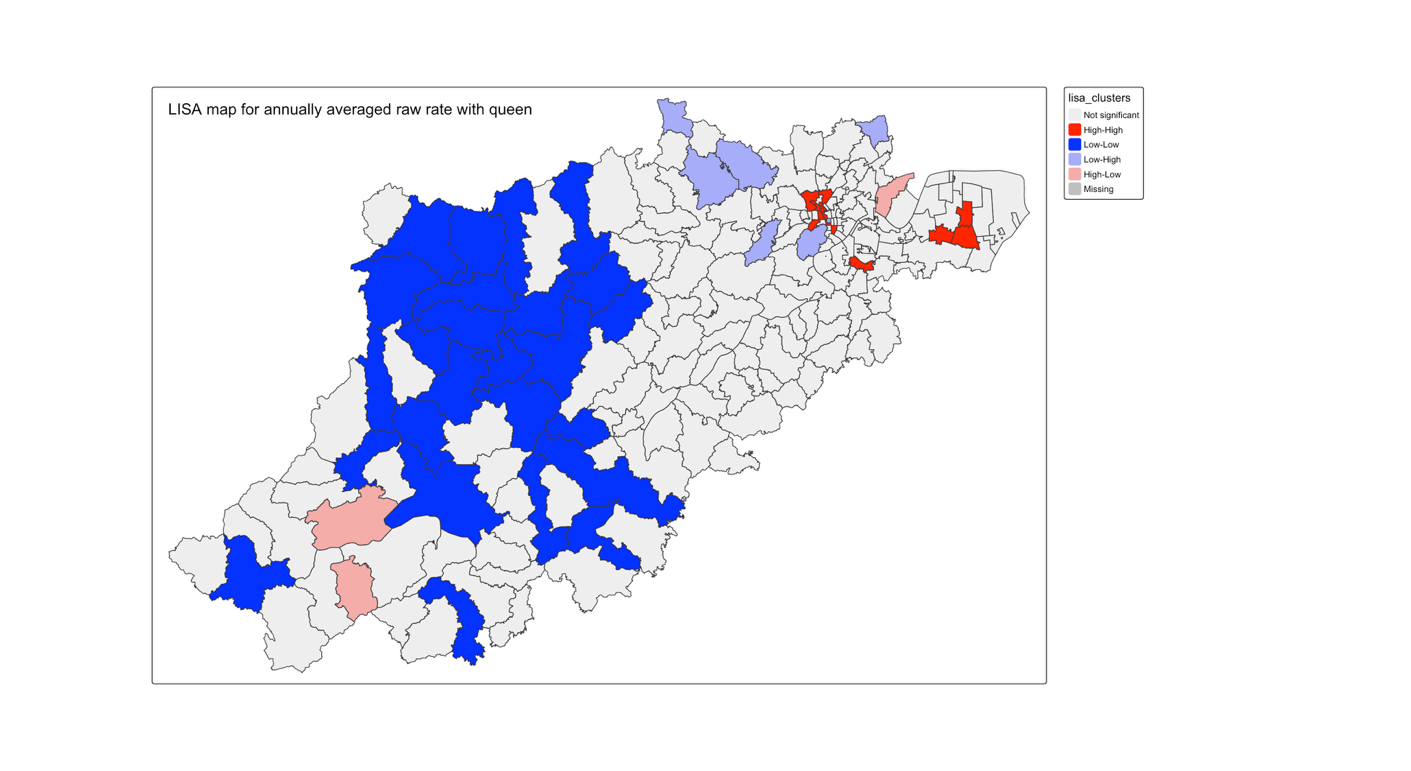
**

**Figure 13. LISA map for annually averaged rate with queen weight**

**
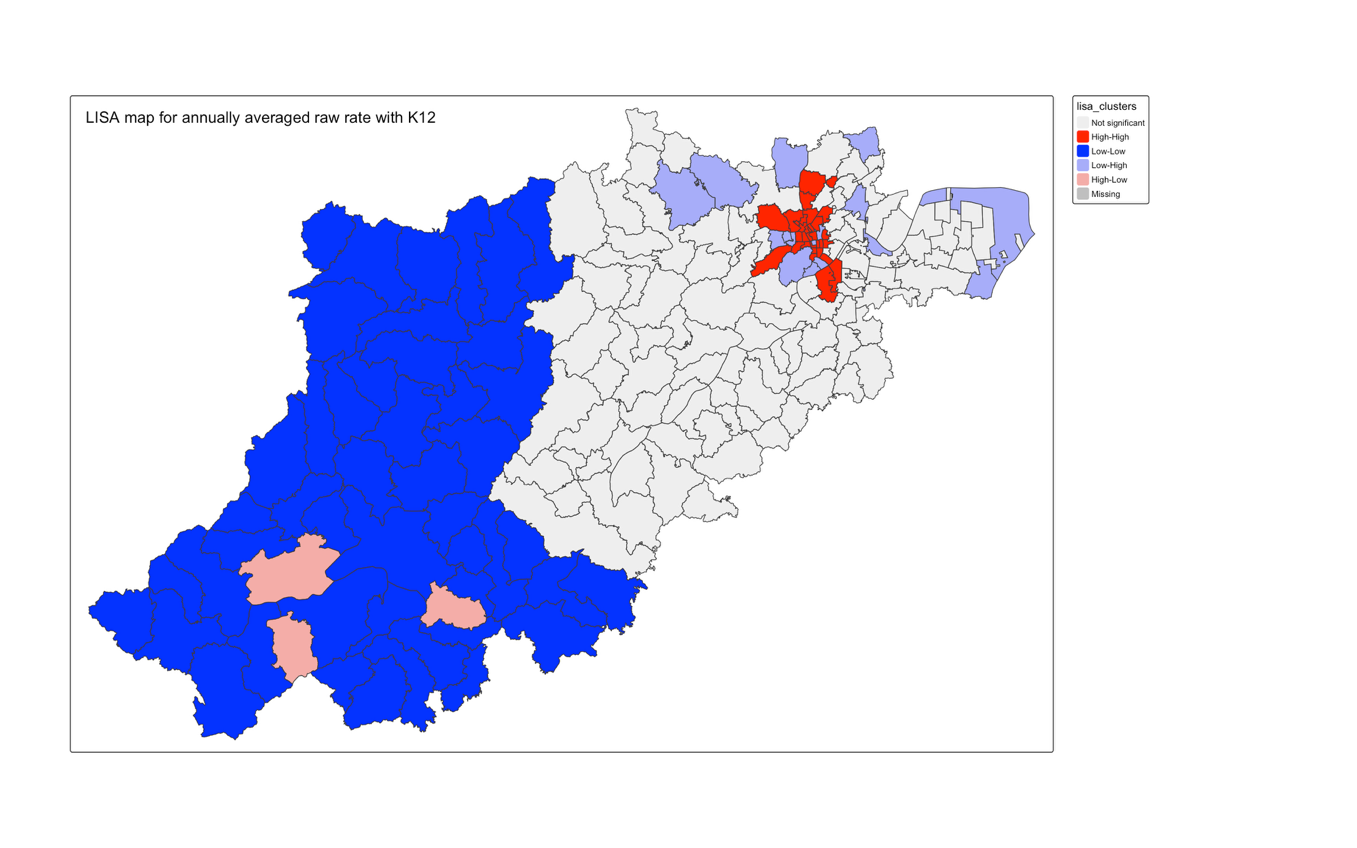
**

**Figure 14. LISA map for annually averaged rate with K = 12**

**
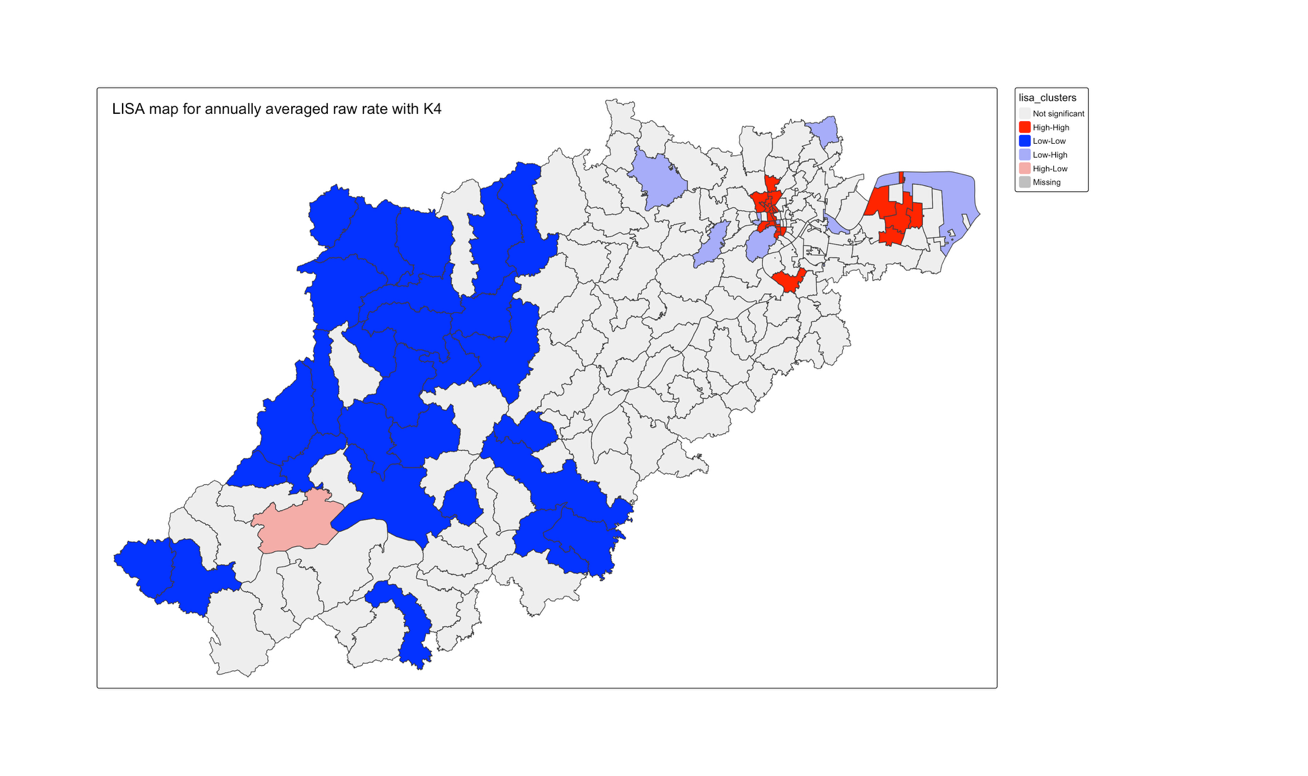
**

**Figure 15. LISA map for annually averaged rate with K = 4**

**
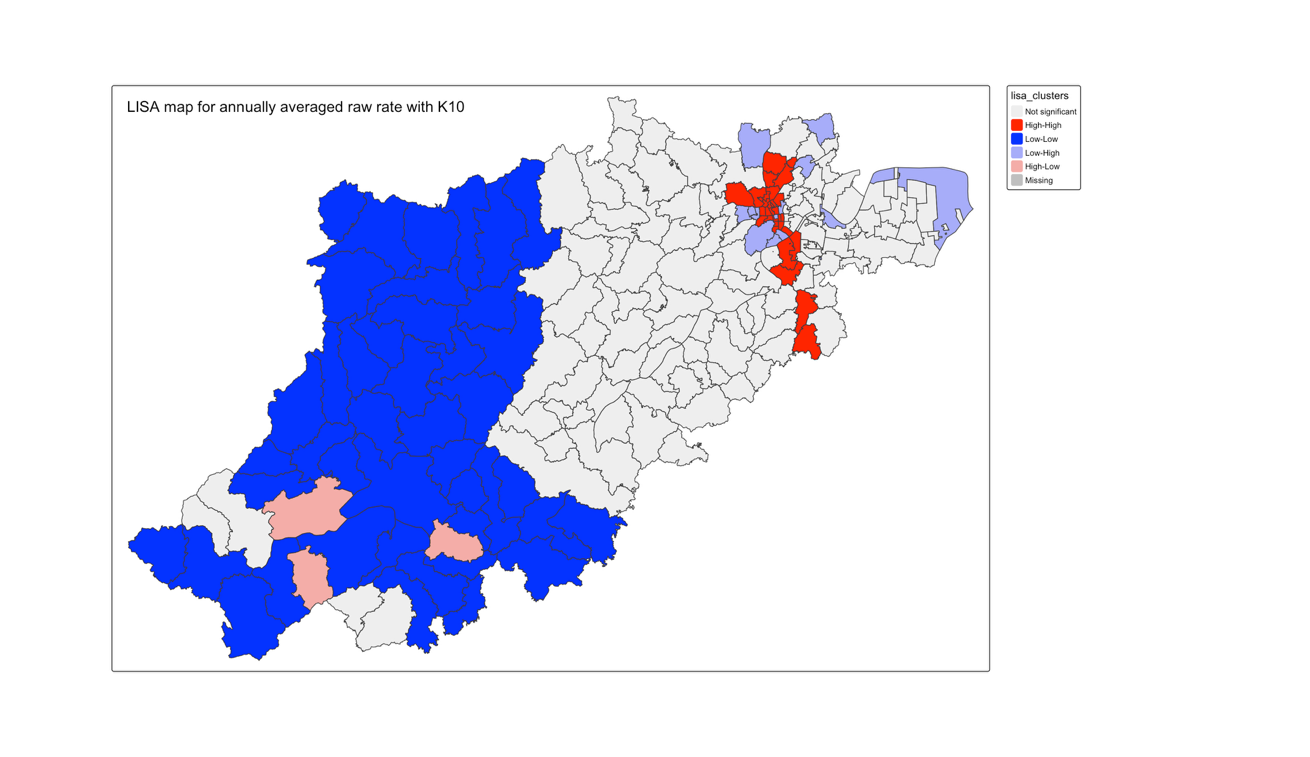
**

**Figure 16. LISA map for annually averaged rate with K = 10**

**
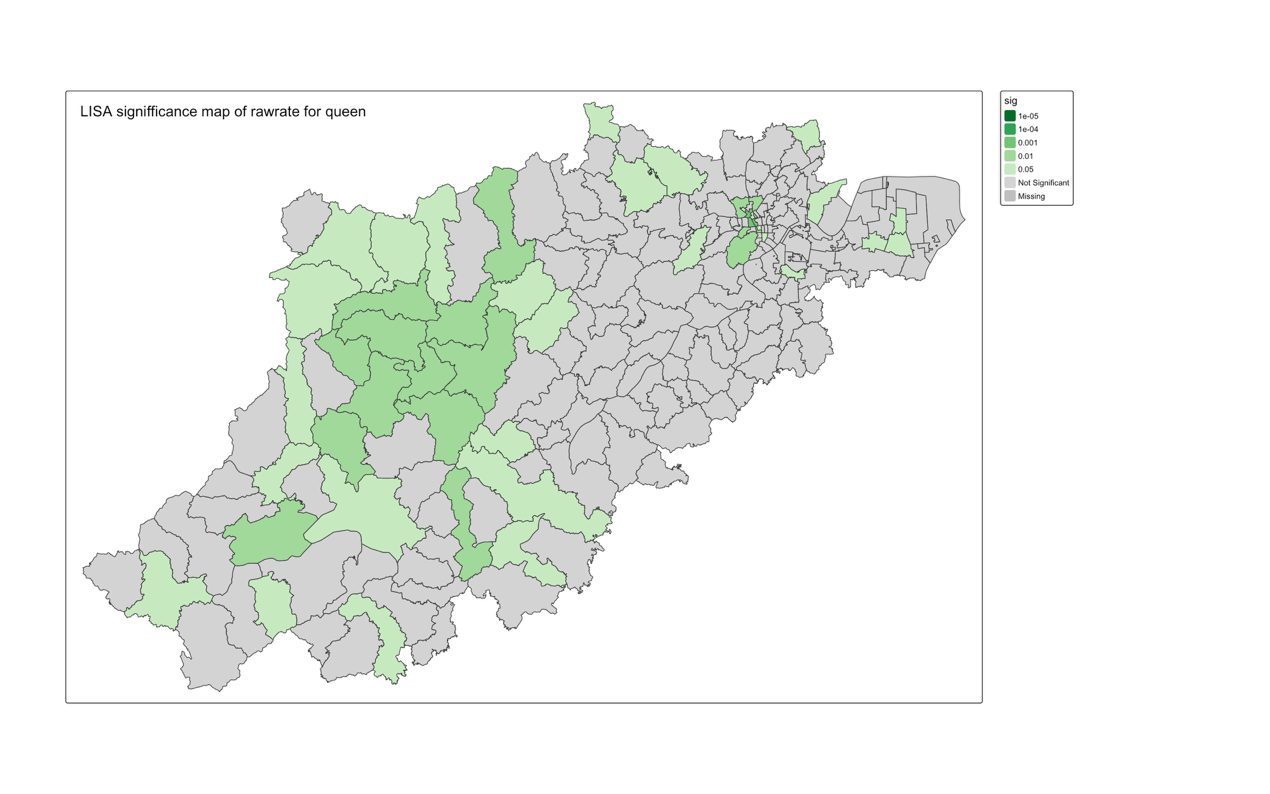
**

**Figure 17. LISA significance map for annually averaged rate with queen weight**

**
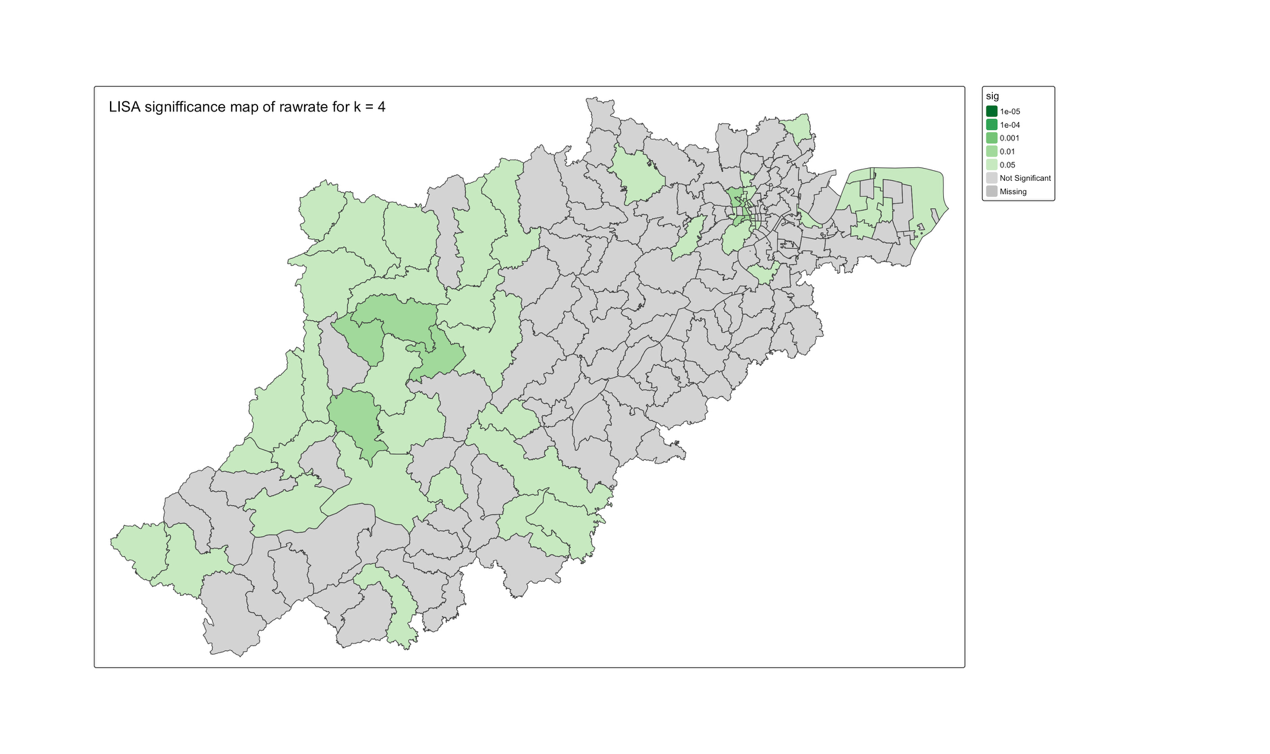
**

**Figure 18. LISA significance map for annually averaged rate with K=4**

**
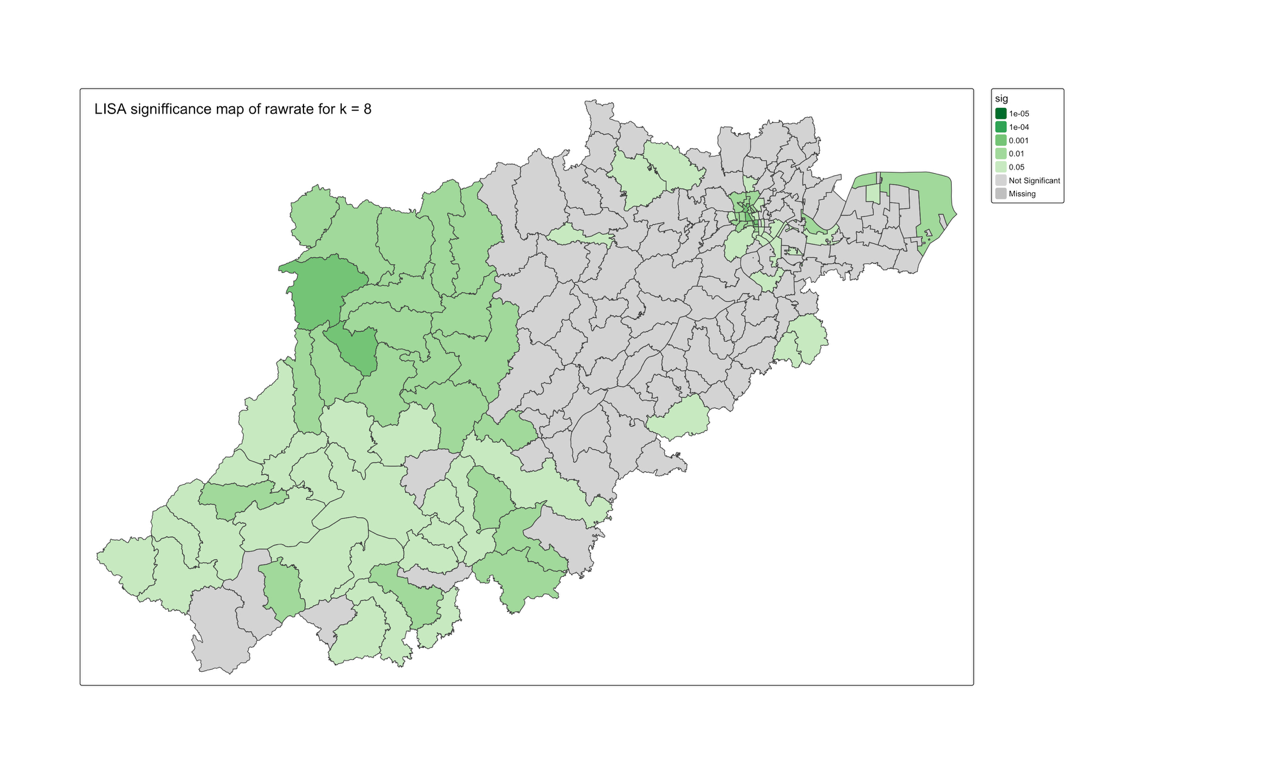
**

**Figure 19. LISA significance map for annually averaged rate with K=8**

**
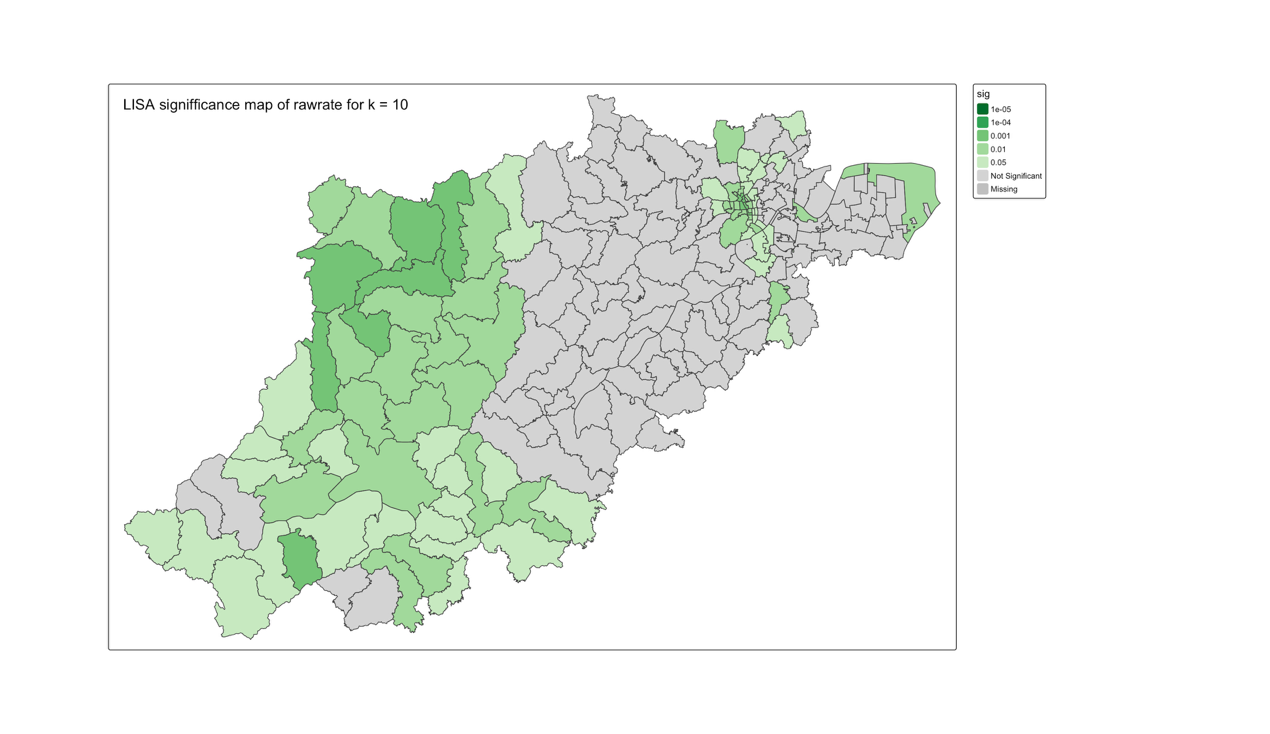
**

**Figure 18. LISA significance map for annually averaged rate with K=10**

**
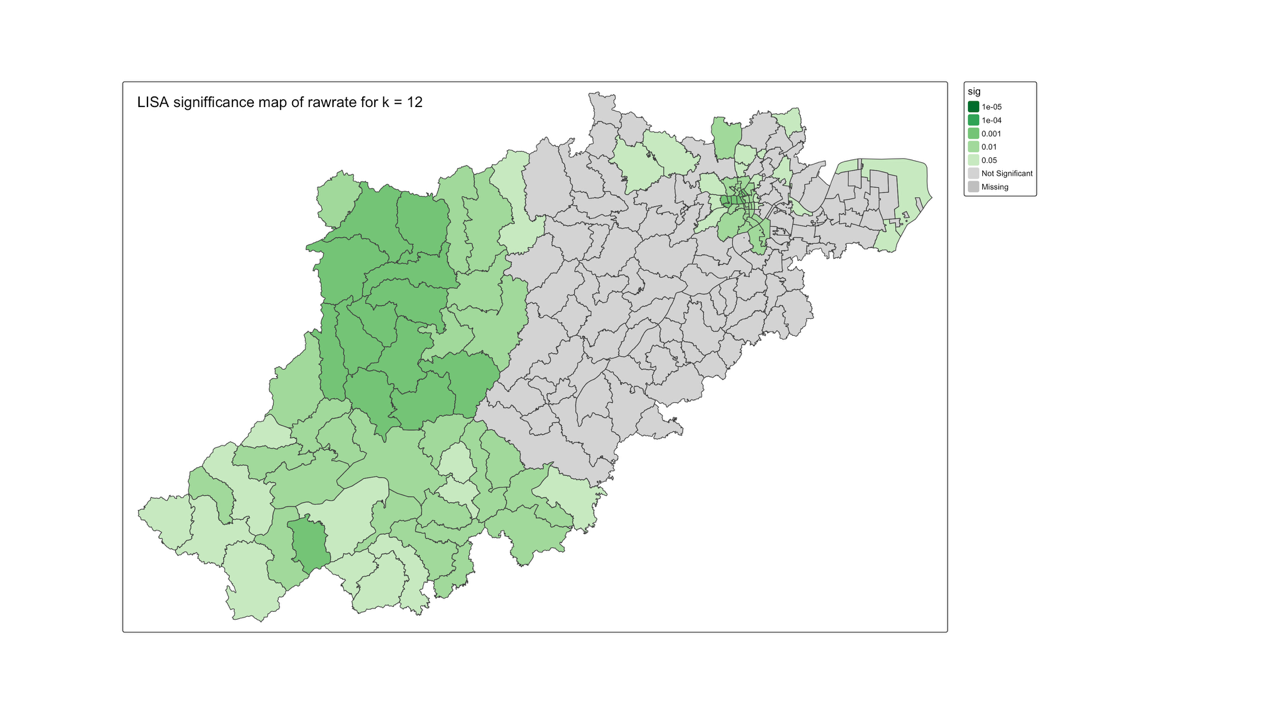
**

**Figure 18. LISA significance map for annually averaged rate with K=12**
